# First evidence for cattle traction in Middle Neolithic Ireland: A pivotal element for resource exploitation

**DOI:** 10.1371/journal.pone.0279556

**Published:** 2023-01-26

**Authors:** Fabienne Pigière, Jessica Smyth

**Affiliations:** School of Archaeology, Newman Building, University College Dublin, Belfield, Dublin, Ireland; University of California Santa Cruz, UNITED STATES

## Abstract

The power harnessed by cattle traction was undeniably a valuable asset to Neolithic communities. However, data are still lacking on the timing, purposes, and intensity of exploitation of draught animals. This paper sheds new light on a region of Europe–Neolithic Ireland–for which our knowledge is particularly restricted as evidence from both Ireland and Britain in this period has been so far patchy and inconclusive. Using a suite of methods and refined criteria for traction identification, we present new and robust data on a large faunal assemblage from Kilshane, Co. Dublin that strongly support cattle traction in the middle 4th millennium BC in Ireland. Bone pathology data combined with osteometric analysis highlight specialised husbandry practices, producing large males, possibly oxen, for the purpose of cattle traction. This new technology has important implications for early agriculture in the region since it provides a key support for more extensive land management practices as well as for megalithic construction, which increased considerably in scale during this period. We argue that access to draught animals and the exploitation of associated resources were at the heart of wider changes that took place in Neolithic Ireland in the second half of the 4th millennium BC.

## Introduction

Like other elements of the Secondary Products Revolution promoted by Sherratt for Neolithic Europe [1–3], the exploitation of cattle for labour has been reassessed in recent decades and his model of a unique innovation horizon including transfer of know-how from the Near East to Europe has been critiqued on both theoretical and empirical grounds [4–14]. In particular, the lack of zooarchaeological evidence to support the idea that animals were initially raised for meat and only subsequently exploited for secondary products (milk, wool, and traction) has been highlighted.

Recent regional analyses of the adoption of cattle traction in Southwest Asia and Europe have been performed based on multi-proxy approaches that include new osteological evidence and methodological improvements [4–11]. These studies argue that specific socio-economic contexts were crucial drivers for the acquisition of this technological innovation, resulting in different timings, reasons, and processes for the adoption of cattle traction according to the region and societies involved [6, 8, 11].

In Southwest Asia, the use of cattle for work, most likely for draught and transport, appears to begin during the Middle PPNB, c. 8200–7500 cal. BC, and castration seems attested during the same period in Syria (at Tell Aswad) and in Turkey (at Cafer Höyük) [10, 11]. In the Western Mediterranean, the occasional exploitation of cattle for the pulling of heavy loads has been argued as early as the onset of the Neolithic at La Draga in Catalonia c. 5300–4900 cal. BC, while in the Balkans, the first evidence of occasional use of cattle for work is dated to c. 6000 cal. BC [9, 10]. In northwest Europe, the earliest evidence for draught cattle is provided by exceptional waterlogged finds from Scandinavia and Switzerland dated to the 4th millennium BC, the Middle Neolithic. In southern Scandinavia, a cattle skeleton found in a bog and dated to 3650–3360 cal. BC displays pathologies on the metatarsals that can be related to traction [6], while in Switzerland, the Arbon-Bleiche 3 yoke, dated to 3384–3370 cal. BC, is the earliest evidence of the use of a pair of cattle for traction [7]. Neolithic sacrifice of work cattle has been evidenced through the burial of a pair of draught oxen harnessed to a wagon from Profen in Germany [15].

This paper focuses on the northwest Atlantic islands for which the exploitation of cattle traction remains largely unknown. In southern Britain, the use of the ard is documented by criss-cross furrows underlying the mid-4th millennium BC South Street long barrow in Wiltshire [16]. It has also been suggested that osteoarthritis, a degenerative joint disease, recorded on pelvises and scapulae from cows at the Etton causewayed enclosure in Cambridgeshire may be linked to the use of the animals for traction [17]. However, the aetiology of this pathology is complex and other causes, such as aging, trauma or infectious disease, appear to play a more preeminent role [6, 18, 19]. Moreover, osteoarthritis has not been recorded on the scapulae of modern draught cattle [19, 20], suggesting that another factor was responsible for the Etton pathologies.

**Fig 1:**
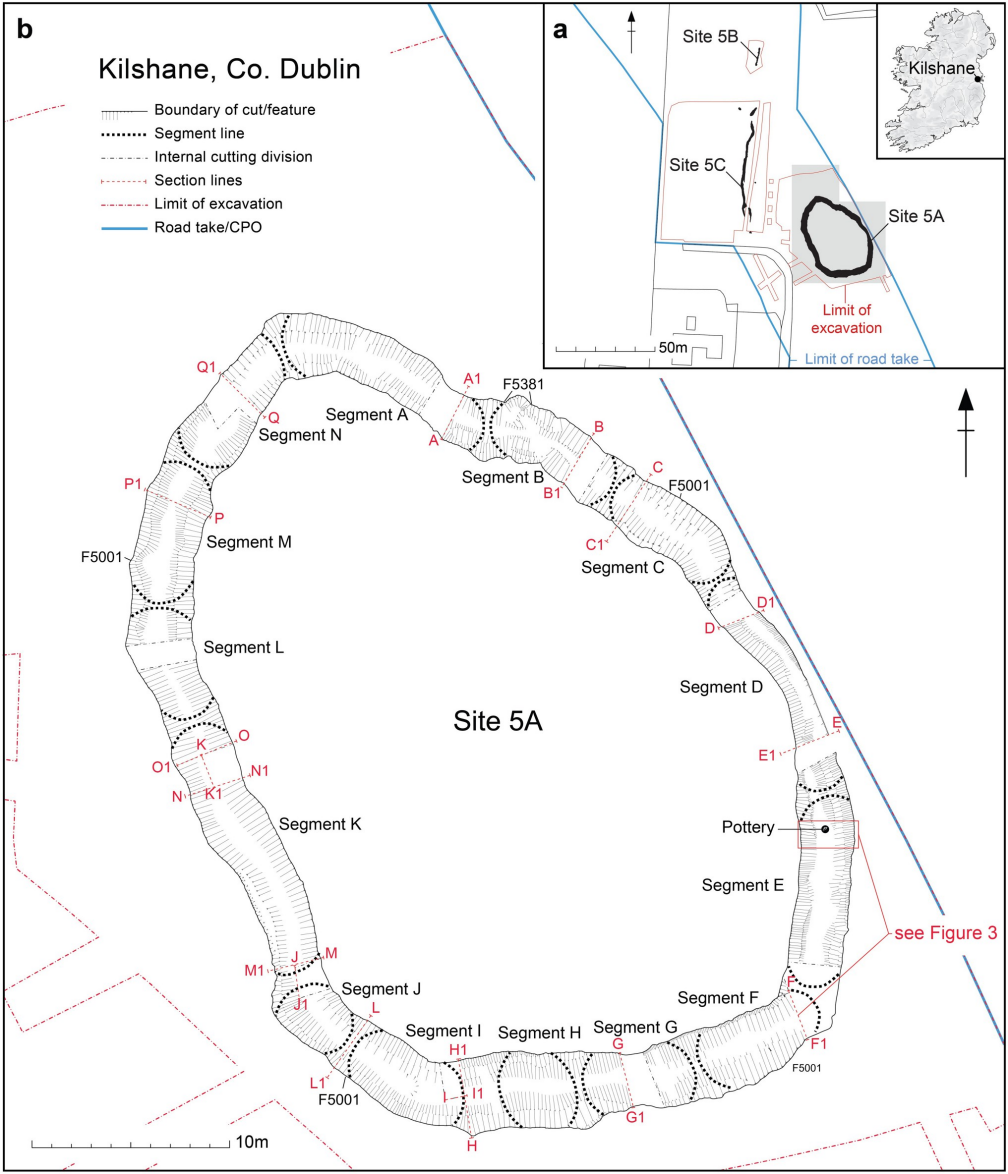
Extent of excavated areas at Kilshane, Co. Dublin, with (a) site location and (b) the excavated enclosure ditch Site 5A.

**Fig 2:**
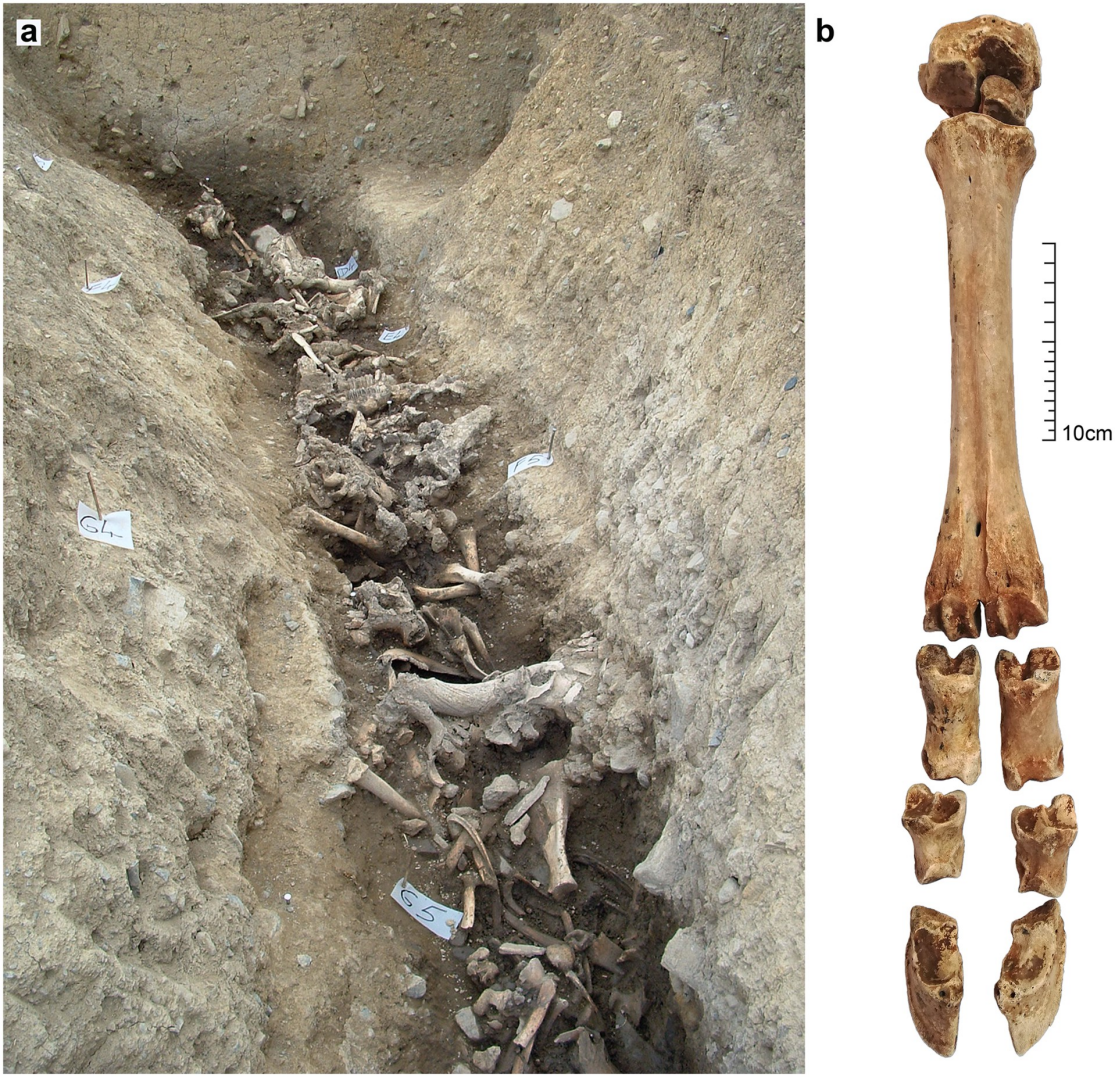
(a) View of *in situ* cattle bone deposit in Segment D of the Kilshane enclosure ditch (photo courtesy of Transport Infrastructure Ireland) and (b) articulated foot bones recovered from F5418, part of the cattle bone deposit within Segment D of enclosure ditch F5001.

While prehistoric bone assemblages are generally poorly preserved in Ireland [21], the bone assemblage from the later 4^th^ millennium BC enclosure at Kilshane, Co. Dublin [22] (Figs 1 and 2), represents an exceptional survival and an important ’window’ on Neolithic animal husbandry. The cattle bone assemblage from Kilshane, representing a minimum of 58 cattle individuals, provides good conditions to investigate husbandry practices and specifically to assess the exploitation of cattle for traction due to the large collection of mostly complete bones from all parts of the skeletons and crucially a high number of foot bones (metapodials and phalanges) on which the study of pathologies related to traction focuses [20, 23]. The relative diversity in the kill-off patterns of cattle at this site indicates that individuals selected for feasting were not solely bred for this purpose [22]. While a high proportion of cattle were slaughtered at the optimum age for meat production, at about 2 years of age, there was also a significant number of adult cattle over 40 months and among them a small group older than 6 years. These adult individuals could have been kept for milk production, as breeding stock or to be used for traction. Lipid analysis of pottery vessels confirms that dairying is practised from the start of the Neolithic in Ireland, in the early 4th millennium BC, and milk remains an important product into the 3rd millennium BC [24, 25]. However, the possible exploitation of cattle traction has not to date been investigated Assessing the use of cattle for traction is a complex matter as there are no pathologies unequivocally distinctive of traction. To overcome this difficulty, multiple lines of evidence and the latest methods have been combined to investigate the use of cattle for traction in later 4th millennium BC Ireland.

As outlined above, the 4th millennium BC appears to be when this technology was adopted in various parts of northern Europe, making the Kilshane assemblage a key site to further examine this phenomenon. The aim is not only to identify cattle traction but also the intensity of this practice, and if animals may have been bred for the purpose. Finally, potential reasons for the adoption of cattle traction are examined using evidence from zooarchaeology, archaeobotany, and archaeology.

## Material and methods

**Fig 3:**
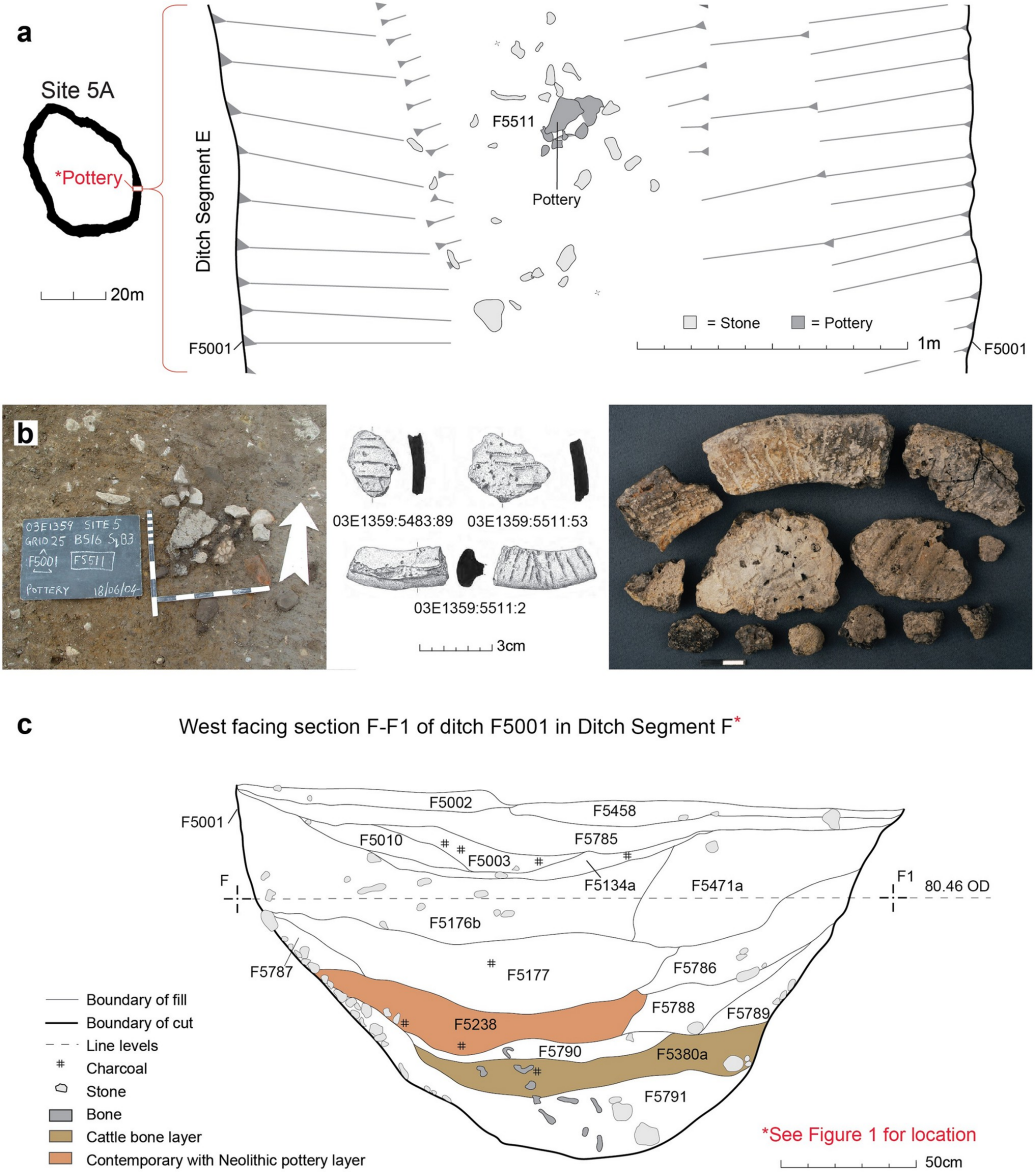
a) Plan of enclosure ditch showing location of Middle Neolithic pottery deposit; b) mid-excavation photo of pottery *in situ*, illustrations and photo of select rim and body sherds; c) stratigraphy of the enclosure ditch showing position of cattle bone layer relative to pottery deposit.

The Kilshane assemblage is composed of 3866 skeletal elements, nearly exclusively cattle bone, derived from the bottom of the segmented enclosure ditch and represent the primary Neolithic deposit (Phase 1B layer; Fig 1) [22]. Both articulated and disarticulated elements were deliberately deposited into the base of the ditch around its full circumference (Fig 2). While there was some suggestion of sequence to the digging of individual ditch segments, the minimal evidence for silting and the continuous spread of cattle bone across some adjoining ditch segments indicate that this deposition took place more or less contemporaneously. The bone layer was sealed by a series of clay and silt deposits around the circumference of the ditch, the result of natural infilling, deliberate backfilling or both. One of these deposits, a compacted silty clay containing quartz, bone and mollusc remains, had lying within it a semi-intact Middle Neolithic broad-rimmed globular bowl, interpreted by the excavator as a deliberately placed vessel (Fig 3) [26]. Directly overlying this was a grey clay deposit containing charcoal (probably *Fraxinus* sp.) dated to 3650–3510 cal. BC (Wk-18170; 4784 ± 33 BP) at 95% probability using OxCal v 4.4.4 [27] and the IntCal20 calibration curve [28]. Attempts to directly date the cattle assemblage was performed first via radiocarbon dating of dental and bone collagen and then via single compound dating of bone lipids, however both methods proved unsuccessful due to low collagen and lipid yields [22]. To conclude, stratigraphic and chronological data indicate a single deposit dated to the Middle Neolithic, perhaps as early as 3600 cal. BC.

Two methods were used to study pathologies related to the draught use of cattle. Firstly, we applied the method described by Bartosiewicz et al. [20], which is based on an osteological study performed on a modern population of cattle that had been used all their life as draught animals in the Carpathian mountains. In addition, Bartosiewicz et al. also analysed fattened bulls raised only for meat as a reference assemblage for cattle never used for traction. This work led to the definition of a series of draught-related anomalies on the foot bones (metapodials and phalanges). The present study focuses on the phalanges, for which five pathological/sub-pathological deformations have been defined: proximal and distal exostosis, proximal lipping and osteoarthritis on the proximal and distal articular surface [20, 29]. All pathologies are described in a quantitative way, using a scoring system ranging from 1 to 4 for the exostoses and lipping [20, 30]. A score of 1 corresponds to an absence of pathology and the increasing development is expressed by scores 2–4 [29]. Osteoarthritis is recorded as present (score 1) or absent (score 2).

Different factors (age, sex, live weight and draught or traction work) can also cause pathologies/sub-pathologies on cattle bones. Recent pathological studies on aurochs’ skeletons and a semi-feral herd from Chillingham Park (UK) indicate that pronounced distal exostosis on the first phalange can be associated with the advanced age of animals [5, 6, 9, 19], however no similar link has been found with the development of articular surface extensions (lipping). The latter pathology is extremely common on the feet of modern draught cattle resulting from an adaptive remodelling of overloaded joints [5, 20, 29]. Similarly, proximal and distal exostoses, as well as proximal lipping, on the second phalange are not significantly influenced by age, whereas these pathologies on the third phalange are positively correlated with the age of animals [19].

The selection of bones and the specific pathologies to be analysed were based on the results of these previous studies as well as the state of preservation of the Kilshane material. Analysis focused on the first phalanges, which were better preserved compared to the second phalanges. Due to poor preservation conditions in the soil, the surface of the second phalanges were frequently highly eroded not allowing us to perform a pathological analysis on a large enough assemblage. As much as possible the analysis targeted only one anterior and one posterior phalange from the same individual. In total 85 phalanges were studied. Anterior and posterior first phalanges were analysed separately, using the criteria provided by Dottrens [31] to distinguish them. In order to assess the causes of pathological development on the cattle bones, the pathologies/sub-pathologies were first examined individually and compared with the pathological analysis performed on 44 aurochs’ skeletons of Boreal date and on 122 cattle from the Neolithic Funnelbeaker culture (TRB) in southern Scandinavia [6]. These cattle bones are dated to the Early Neolithic (4000–3300 cal. BC), the Middle Neolithic I (3300–3100 cal. BC) and the Middle Neolithic II-V (3100–3000 cal. BC).

In addition, a pathological index was calculated for 29 anterior and 37 posterior first phalanges using the pathological index formula based on five pathologies: proximal and distal exostoses, proximal lipping and osteoarthritis on the proximal and distal articular surface [20, 29]. A modified pathological index omitting distal exostosis (strongly influenced by the animal’s age) was also calculated for the same phalanges [19]. The mean pathological index obtained on the Kilshane assemblage was compared with those from modern reference specimens, namely oxen used all their life as draught animals in the Carpathian mountains [20], Romanian young bulls fattened for meat production [20] and semi-feral Chillingham cows and bulls [19]. Where possible, comparisons with reference specimens were made separately for anterior and posterior phalanges. However, for several collections no separate analysis was carried out or the study focused either on the forelimbs or on the hindlimbs.

A second method, designed by Lin et al. [23], was applied on metapodials. It consists of the calculation of an index (e/D1), using measurements taken on the distal metacarpal and metatarsal. This index describes the degree of remodelling and extension of the medial condyle of the metapodials. A value of 0.75 or higher indicates a degree of remodelling consistent with traction, which is based on comparisons made with modern traction animals and aurochs [9, 23]. The modern traction references include 18 Romanian Grey and Brown castrated males and 3 Jersey oxen [23]. Male and female aurochs dated to the Neolithic period (c. 6100/6000–4500 cal. BC) are from the central and south Balkans [9]. A recent study has demonstrated that the broadening of the metacarpal is strongly correlated with age, while no such correlation has been established for the same pathology on the metatarsal [19]. Studies of cattle herds intensively used for traction present more severe pathologies on the hindlimbs than on the forelimbs [19]. This appears to reflect the fact that draught work requires significantly increased retrorse thrust. Repeated and increased loading on the hindlimbs results in adaptive remodelling to strengthen and stabilise joints, which eventually impacts the bone tissue. Based on these results, the index calculated on the metatarsal appears to be a more reliable identifier of traction while biological variables, age in particular, strongly impact the same modification on the metacarpal. This second method offers a more objective assessment of the severity of pathologies as the scoring system of the first method introduces a non-negligible element of subjectivity in the attribution of the score [9, 23].

Our analysis also includes age-at-death, sex-ratio, and osteometric data to examine the different factors responsible in the development of pathologies and whether cattle selected to be used as draught animals responded to specific demographic and physical characters. Cattle age-at-death estimation was based on the eruption and attrition of mandibular teeth following Grant’s method [32] and using the absolute ages provided by Jones and Sadler [33]. The proportion of each age category was calculated based on the minimum number of individuals.

Sexing is based on osteometric analysis of the metapodials and measurements of bones were carried out following the guidelines of von den Driesch [34]. The expression of sexual dimorphism on these bones has been documented by studies on modern cattle populations [19, 35–37], which demonstrate a stronger sexual dimorphism expression on the metacarpal than the metatarsal. They show that males have more robust metapodials than females, while males castrated at a young age can be distinguished from bulls and cows by their taller stature, also resulting in a relative higher gracility. Here we complement a previously published sexing approach based on width measurements of distal metacarpals (Bd) [22] with a method using a gracility index on the smallest breadth of the metapodial diaphysis (SD/GLx100) plotted against the greatest length measurements (GL) [37]. To examine size variations among Neolithic cattle, the withers height of cattle was calculated on the greatest length of metapodials, by using factors provided by Matolcsi [38].

## Results

### Analysis of cattle phalanges

**Fig 4:**
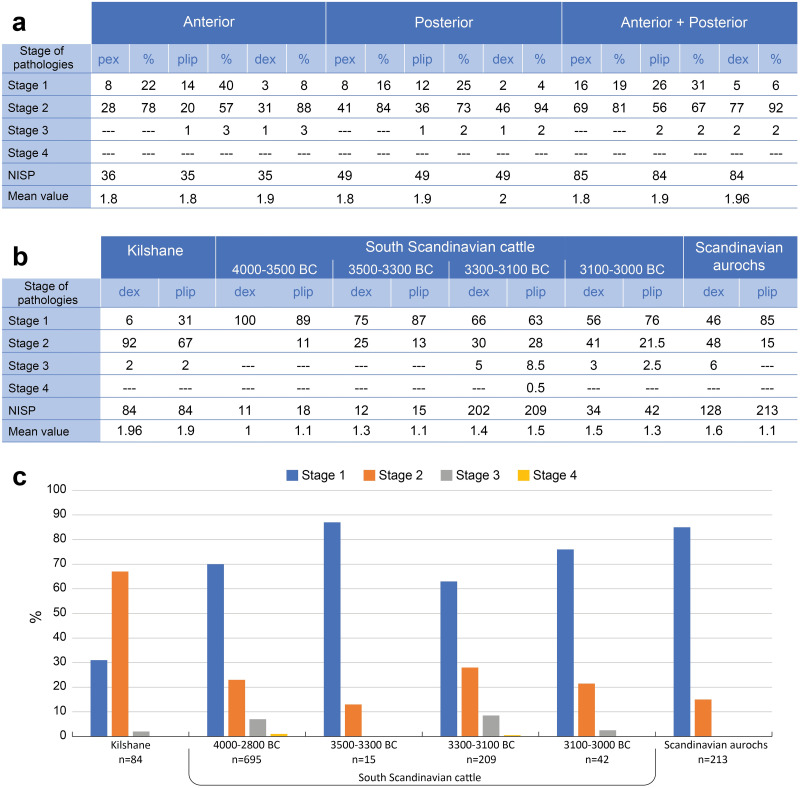
(a) Pathologies on the first anterior and posterior phalanges from Kilshane enclosure; pex, proximal exostoses; plip, proximal lipping; dex, distal exostoses. (b) Pathologies on the first phalanges (anterior and posterior combined) from Scandinavian aurochs, Early and Middle Neolithic Scandinavian cattle (Johannsen 2006) and the Middle Neolithic cattle from Kilshane enclosure; plip = proximal lipping, dex = distal exostoses. (c) Pathologies on foot bones: proximal lipping on the first phalanges (anterior and posterior combined) from Scandinavian aurochs, Early and Middle Neolithic Scandinavian cattle (Johannsen 2006) and the Middle Neolithic cattle from Kilshane enclosure site. Stages of pathologies according to scoring system by Bartosiewicz et al. 1997.

**Fig 5:**
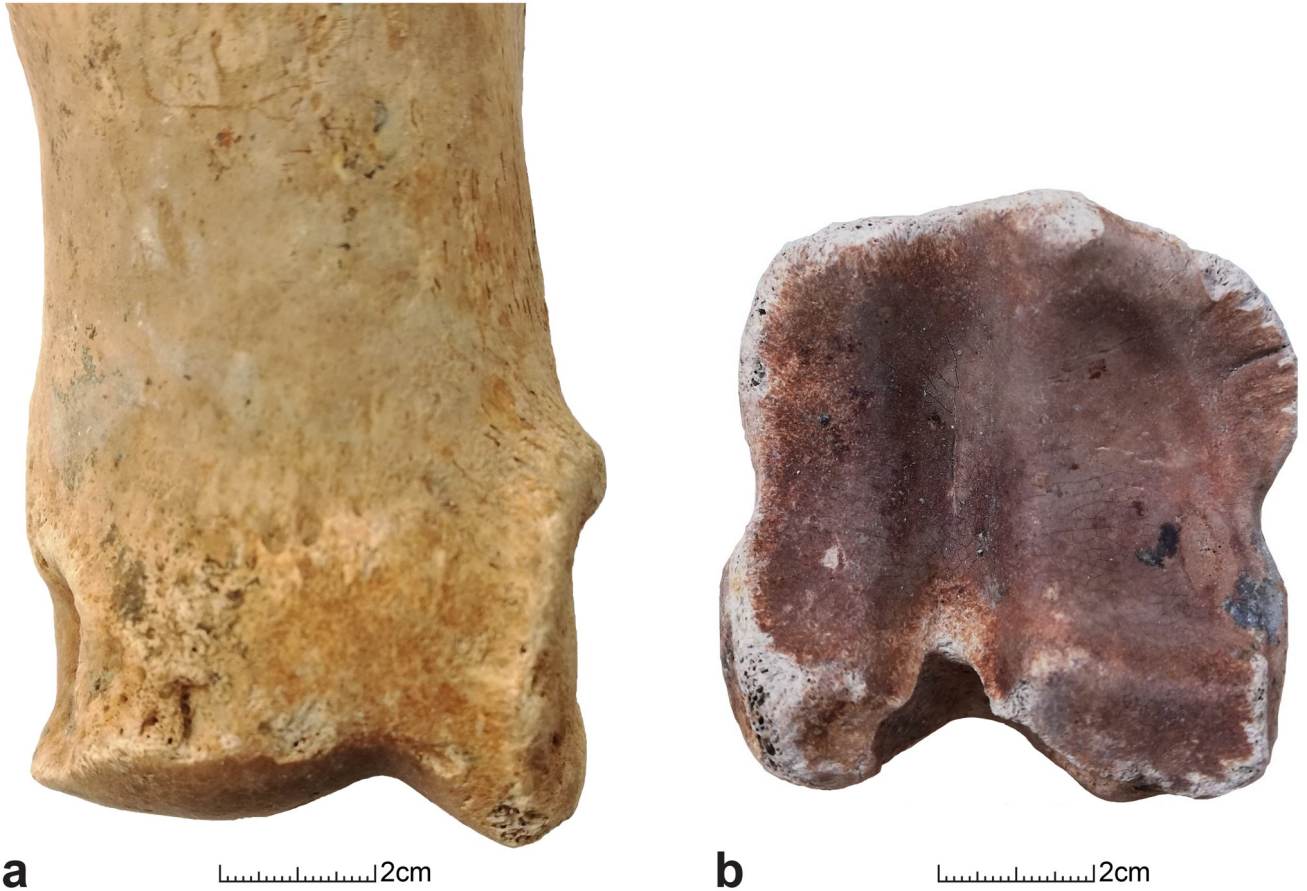
(a) First phalange exhibiting Stage 2 distal exostosis and (b) first phalange exhibiting Stage 3 lipping (stage of pathologies according to scoring system by Bartosiewicz et al. 1997).

A high number of phalanges show a certain level of pathological alteration (Fig 4), with a majority of individuals presenting stage 2 of proximal and distal exostoses as well as proximal lipping (Fig 5a). Rare specimens present an intense pathological remodelling. Only one occurrence of stage 3 distal exostosis and proximal lipping was recorded on an anterior and a posterior phalange (Fig 5b). No osteoarthritis was recorded on the Kilshane cattle bones.

Comparisons with aurochs and domestic cattle dated to 4000–3000 cal. BC from southern Scandinavia show higher incidence of pathological changes on the Kilshane cattle bones [6] (Fig 4b). Indeed, the majority of Scandinavian individuals do not have any exostoses and lipping. While a small proportion of aurochs show stage 3 of distal exostosis, no serious lipping is recorded on aurochs’ bones. On the other hand, lipping is intensively developed on a small group of domestic cattle and related to their use as draught animals (Fig 4c). There is an increase in pathology on Scandinavian cattle bones over time and it is not before the later 4th millennium BC that domestic cattle are considered to be exploited for traction. At Kilshane, the pathologies are slightly more pronounced on the posterior phalanges than on the anterior ones (Fig 4a). On animal skeletons not used for traction (aurochs and the semi-feral herd from Chillingham Park, UK), pathologies are more developed on the anterior phalanges.

**Fig 6:**
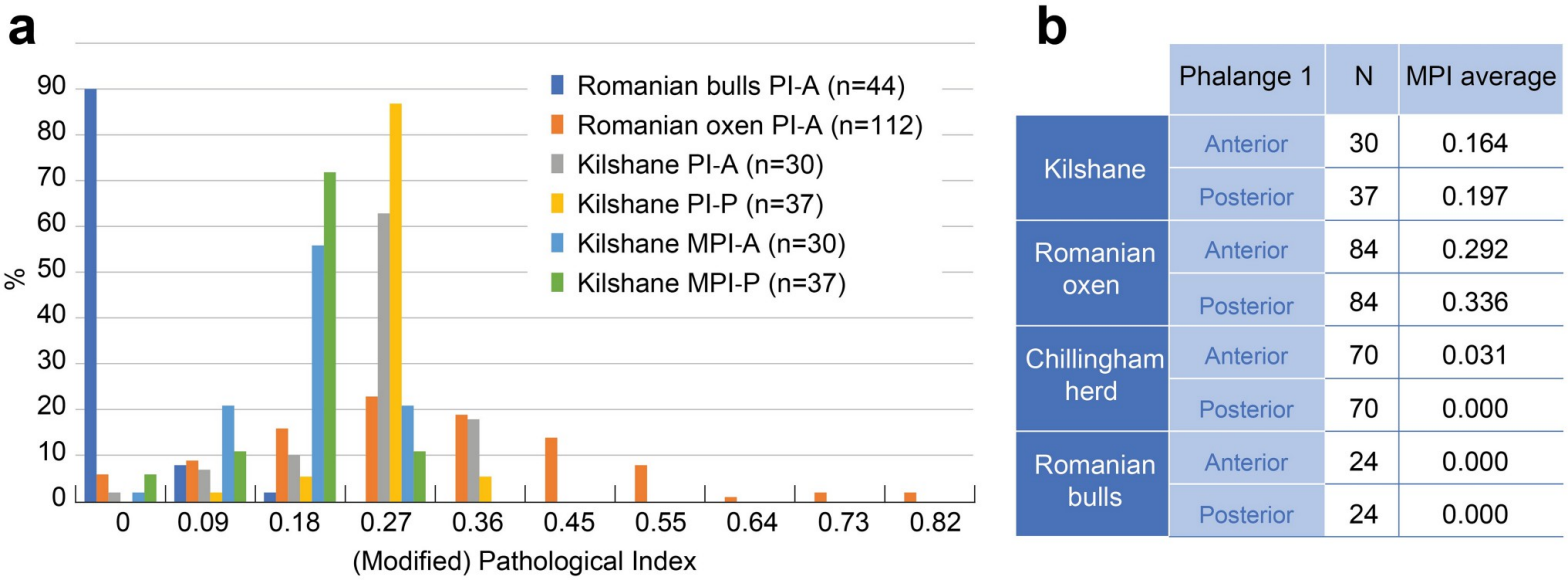
(a) Pathological index on first phalanges from modern Romanian young bulls and draught oxen and the Middle Neolithic cattle from Kilshane enclosure. Data on Romanian bulls and oxen from Bartosiewicz et al. 1997 (b) Mean of the modified pathological index for the first phalanges. Data on Romanian oxen and bulls and the Chillingham herd from Kamjan et al. 2022. A, anterior; P, posterior; PI, Pathological index; MPI, Modified pathological index; N, number of specimens.

The pathological index and the modified pathological index calculated for the first phalanges are presented in Fig 6. The modified index provides lower values (anterior: 0.164, posterior: 0.197) than the unmodified index on average values (anterior: 0.255, posterior: 0.265). When we compare the pathological distribution on phalanges with reference collections of modern Romanian draught cattle and fattened bulls, the Kilshane assemblage displays higher pathological states than the bulls, which can be expected as these latter are very young individuals specifically raised for meat. The Kilshane individuals show a distribution similar to draught cattle, although with a less severe pathological state. Moreover, the modified pathological indices also point out that posterior first phalanges display more serious pathologies than the anterior ones as for the modern draught cattle, while it is the opposite for the young bulls and the Chillingham herd not used for traction.

### Analysis of cattle metapodials

**Fig 7:**
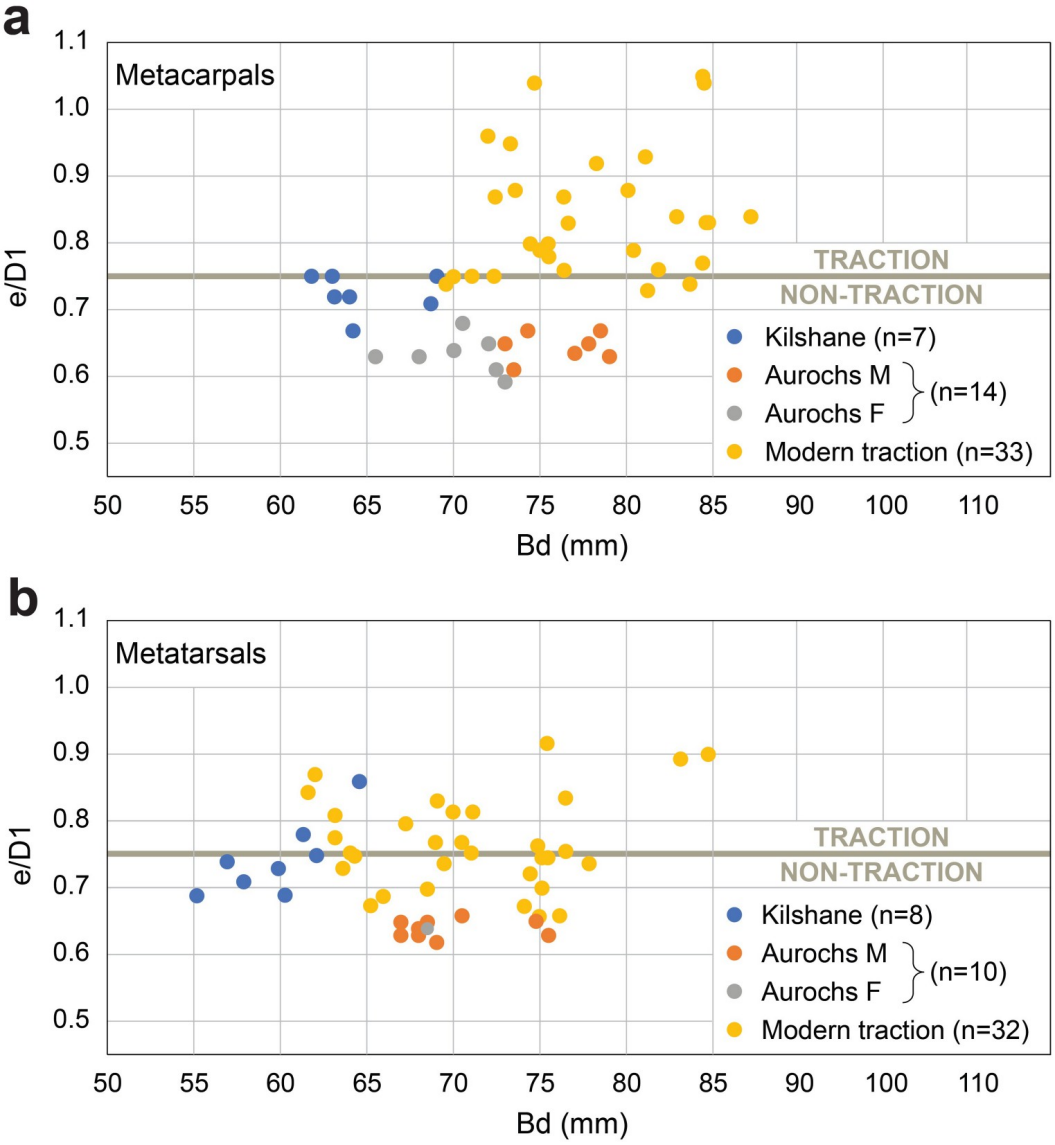
Pathological index and breadth measurements on (a) metacarpals and (b) metatarsals from the Kilshane cattle, modern traction reference animals including Romanian Grey and Brown castrated males and Jersey oxen (Lin et al. 2016) and aurochs from the central and south Balkans dated to the Neolithic period (c. 6100/6000–4500 cal. BC). Horizontal line indicates zone where the traction population can be separated from the non-traction population. M, male; F, female.

**Fig 8:**
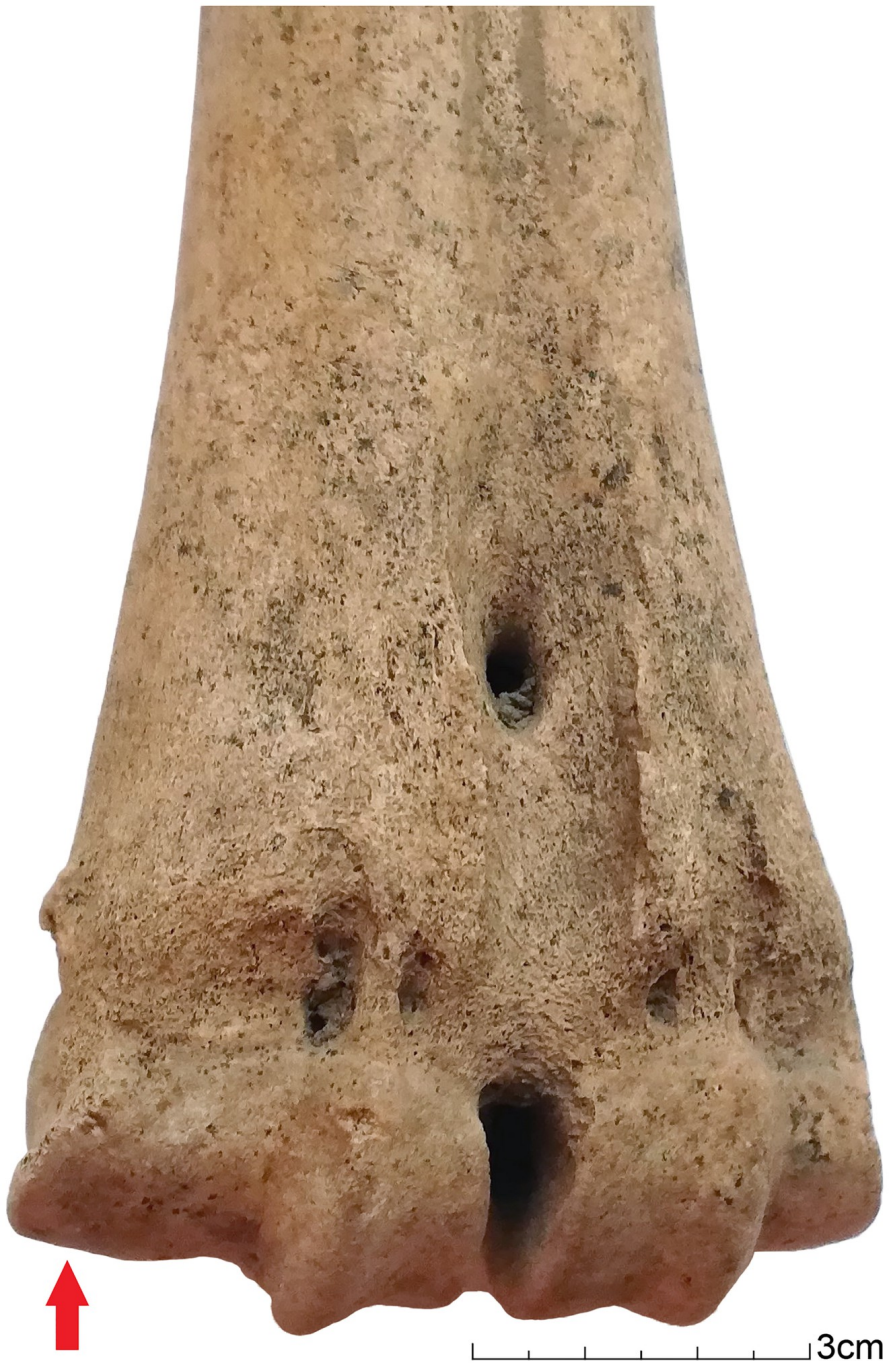
Cattle metatarsal from Kilshane enclosure exhibiting an enlargement of the articular surface. Traction index calculated following Lin et al. (2016): e/D1 = 0.86.

Results of osteometric analysis on distal metapodials from Kilshane, modern draught cattle and aurochs are plotted in Fig 7. Only seven metacarpals and eight metatarsals from Kilshane provided useful data. Such a low number from an assemblage of 58 individuals is due to the numerous young cattle present. Indeed, 80% of the metapodials do not have the distal epiphysis fused to the diaphysis. Kilshane metacarpals and metatarsals present the smallest breadth measurements of the distal ends, gathering on the left side of the graphs, and there are only a few overlaps with the female aurochs. The indices reflecting distal trochlea widening are higher for the Kilshane individuals compared to the aurochs, while two metacarpals and three metatarsals exhibit an index equal to or higher than 0.75 that range within the distribution indices of modern draught cattle (Figs 7 and 8). Overall, higher pathological indices have been recorded for the metatarsals than the metacarpals.

### Kill-off profile, sex ratio and osteometrics

**Fig 9:**
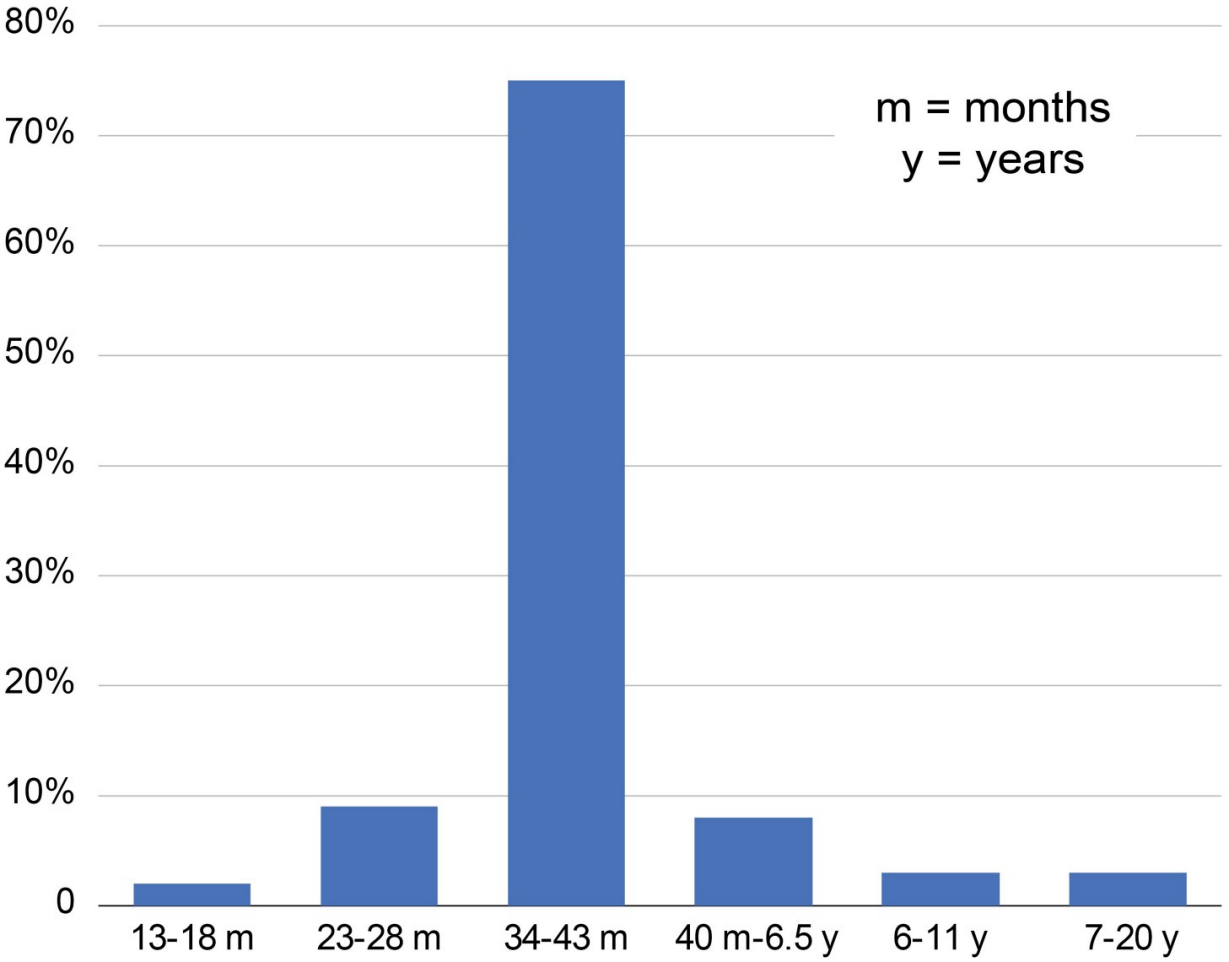
Cattle slaughter distribution at the Kilshane site (MNI = 63). See supplementary material S4 Table in S1 File for raw data. M, months; Y, years.

The kill-off pattern indicates that the majority of cattle were semi-mature individuals killed at the optimum age for meat production around 34–43 months old (74%; Fig 9). However, a small group of cattle were not only bred for meat as they were kept alive over 40 months (14%), and among these 8% were aged between 3.4 and 6.5 years, 3% were 6–11 years old, and 3% were 7–20 years old.

**Fig 10:**
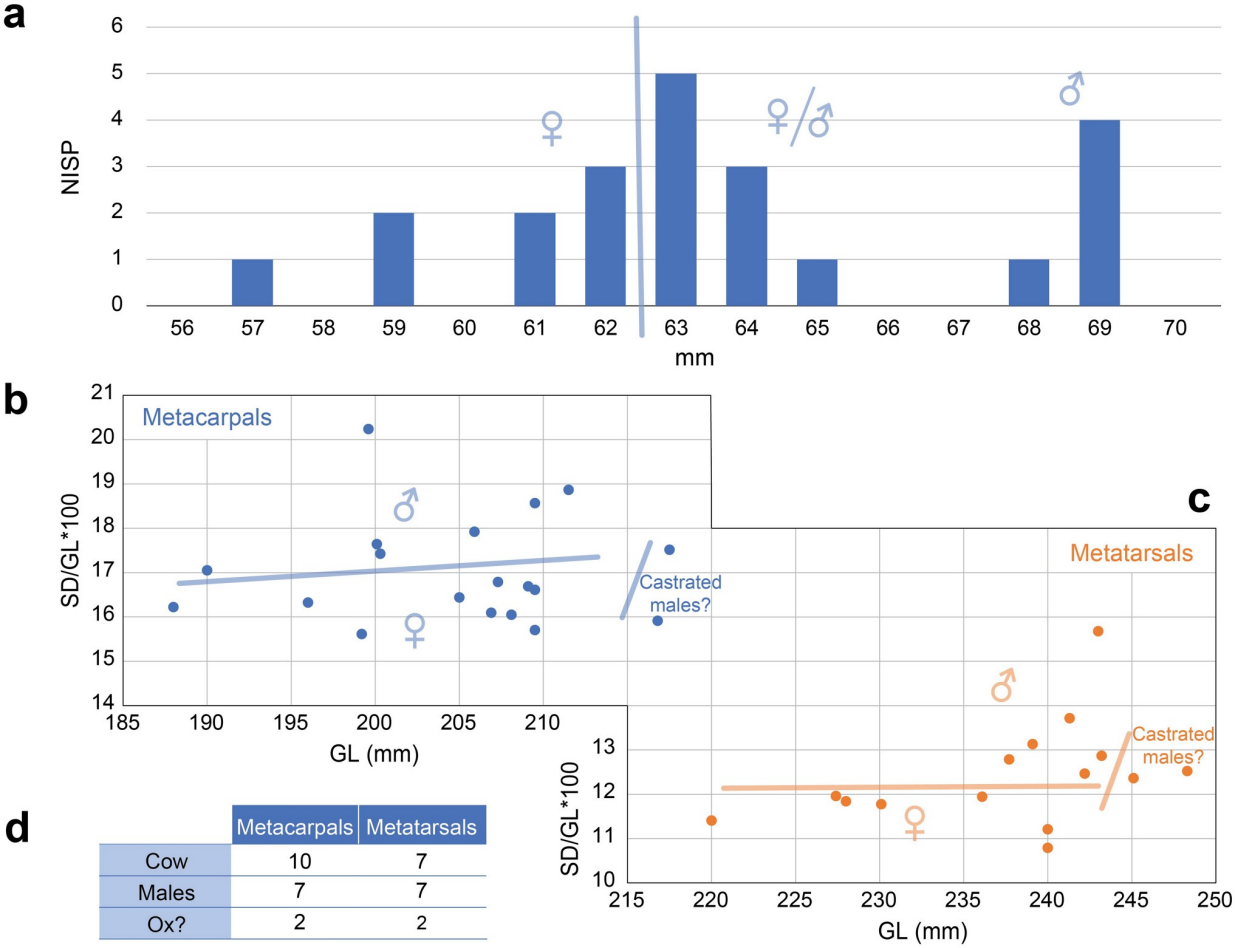
(a) Cattle metacarpal width measurements (Bd) from Kilshane; Distribution of length measurements (GL) plotted against the gracility index of the Kilshane cattle metacarpals (b) and metatarsals (c); (d) Results of sexing Kilshane cattle metapodials using the gracility index.

Regarding sex identification, the plotted distal metacarpal widths of the Kilshane cattle (n = 22) produces three groupings (Fig 10a). Comparisons with larger Neolithic cattle assemblages from Scandinavia and Britain suggest that metacarpals measuring less than 63 mm can be assigned to females [39]. Individuals displaying the largest size, above 65 mm, are likely males, while individuals with measurements between 63 and 65 mm can be from smaller males or larger females. Plotting gracility index on the smallest breadth of the metapodial diaphysis [37] (SD/GLx100) against greatest metacarpal lengths (GL) creates another three groups (Fig 10b). According to the sexual dimorphism criteria stated above, the group presenting the smallest gracility index (less than 17) and the greatest metacarpal lengths (GL) ranging between 188 and 210 cm can be attributed to cows. The greater robustness of the second group (index between 17 and 20) with the same range of GL values as the previous group suggest an attribution of these individuals to bulls. In addition, two individuals stand out from the two other groups by their greatest GL values (above 215 cm) and intermediate gracility index values (index between 15 and 17.5), which can result from the early castration of a male. The sexing pattern provided by the metatarsals (Fig 10c) is similar to that described for the metacarpals and also suggests the presence of two oxen. The results of the gracility index method indicate an equivalent proportion of females and males and therefore seem to confirm that both females and males are represented among the individuals with width measurements between 63 and 65 mm (Fig 10a). This method also suggests the presence of oxen (Fig 10d). However, we need to be cautious due to the small size of the Kilshane sample. A larger measurement dataset from 4th millennium BC Irish cattle is needed to confirm that oxen were bred in Ireland at this point. On continental Europe, it is suggested that castration was practised as early as the mid-6th to early 5th millennium BC [10, 11]. The shoulder height is estimated to complement cattle morphological approach. The sizes calculated on the metacarpals range from 113.7 to 137.6 cm (n = 19), and the values on the metatarsals from 116.1 to 139.5 cm (n = 15).

## Discussion

### Cattle traction and possible breeding of oxen in Neolithic Ireland

Evidence that at least some of the cattle deposited at Kilshane had been used for traction can be argued by the consistent data showing relatively severe pathological development on both phalanges and metapodials. The identification of draught animals relies on pathologies less related to age and sex—proximal lipping and extension of the medial condyle of the metatarsal, which can be better explained by movement resulting from traction [29]. The fact that pathologies are more pronounced on posterior than on anterior bones is further support, as shown by studies on modern oxen used all their life for traction where a stronger strain is observed on the hindlimb than on the forelimb [6, 19, 20]. Conversely, animals never used for traction (aurochs and the semi-feral herd from Chillingham Park) present stronger pathological development on the forelimbs than on the hindlimbs, resulting from natural conditions under which anterior limbs carry a heavier part of the body than posterior limbs. Despite the absence of a comparative sample of aurochs or cattle never used for traction from Irish contexts (to assess the impact of the landscape on pathology development), the clear pathological bias towards the posterior limbs of Kilshane cattle and the high frequency of traction-related proximal lipping pathology makes it unlikely that terrain was the unique and main reason for these modifications and strongly support the traction interpretation.

**Fig 11:**
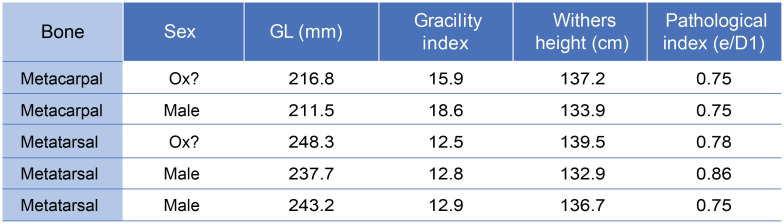
Summary of sex attribution, greatest length measurement (GL), gracility index, withers height and pathological index on the Kilshane cattle metapodials. The high degree of pathology indicates male individuals used for traction.

The metatarsals with a degree of pathology indicative of traction are all from males (n = 5; Fig 11), with two of these individuals possibly oxen, based on metapodial length and robustness. While identification of castrated males using osteometric analysis is tentative, the preference for tall individuals for traction work is clearly reflected in the Kilshane assemblage. We need to take into account that the heavier weight of larger individuals can be responsible for some of the pathological development. However, we argue that weight is not the unique factor. Otherwise, a similar if not stronger pathological state on forelimbs compared to hindlimbs would be observed, which is the opposite here (Figs 6 and 7).

The Kilshane enclosure has been conclusively identified as a site of feasting [22] and this affects our interpretation in two ways: firstly, commensal activities at enclosures likely saw participation from groups of people from different areas [40–42]. We thus need to consider that cattle slaughtered and deposited at Kilshane may have been raised beyond the immediate area–maybe even beyond eastern Ireland–and may have been bred for different purposes. Secondly, as a bone assemblage generated through feasting, the selection of individuals for this specific event probably does not allow a full assessment of the intensity of cattle exploitation for traction. These caveats notwithstanding, we outline below the evidence for activities that may have involved draught cattle in the second half of the 4th millennium BC in Ireland.

### Traction and farming

Evidence for cattle traction is often considered to be connected to agricultural activity, particularly tillage but also the transport of manure. Depending on which use is emphasized, traction can be argued to fit well with both local, intensively cultivated garden plot horticulture and with more extensive fields managed at a distance with a lower input of human labour [43–45]. Garden plot horticulture has been convincingly argued for Early Neolithic Ireland, i.e. the early centuries of the 4th millennium BC, with a higher proportion of annual weed taxa present than Neolithic Britain or central Europe [46]. The earliest clear evidence for cereals appears in the 38th century cal. BC, with emmer wheat predominant and barley playing a secondary role [46–48]. They have widespread occurrence in early 4th millennium BC contexts, although at low levels, which is argued to be consistent with generally small-scale production [46]. Middle Neolithic contexts—those broadly contemporary with Kilshane—remain less well-sampled and under-analysed. The available archaeobotanical data suggest a similar frequency of cereals in the period 3600–3400 cal. BC with a marked reduction from 3400–2500 cal. BC that may signal a shift away from arable agriculture [46, 48, 49]. A similar drop in palaeoecological indicators of arable activity has also been observed [50]. In terms of tillage, there is the single incidence of ard marks recorded at an Early Neolithic house site at Ballygalley, Co. Antrim, in northeast Ireland [51] and a lynchet beneath peat layers dated to 3100–2900 cal. BC at Belderg Beg within the Céide field systems in Co. Mayo in the northwest of the island [52]. While the local, domestic context of Ballygalley reinforces the model of garden plot agriculture, the Belderg Beg lynchet is part of a suite of evidence for more extensive land management at Céide in the later Neolithic [53], something that cattle traction may have precipitated.

### Wheeled transport

Elements from wheeled vehicles have been identified in several places in the Middle East and Europe in the second half of the 4th millennium BC, with recent chronological refinement as well as typological dissimilarities suggesting wheeled vehicles appear simultaneously in both areas [53]. In western continental Europe, wooden wheels and axles from vehicles have been recovered in wetland environments from Switzerland (Vinelz, Zurich), Germany (Waldsee/Aulendorf, Moorweg, Lengener Moor, Profen), and the Netherlands (Eese) [7, 15, 54]. In Britain, a Bronze Age wheel was recovered at Flag Fen [55]. To date, the earliest evidence for wheeled transport in Ireland is an alder block wheel fragment recovered from a Late Bronze Age trackway in Edercloon Bog, Co. Roscommon [56] (c. 1200–970 cal. BC). Prior to this find, the earliest known wheels were a pair of Early Iron Age block wheels recovered from a bog in Doogarymore, also in Roscommon [57, 58] (c. 520–390 cal. BC). With a gap of more than two millennia between the Edercloon wheel fragment and the Kilshane evidence for cattle traction there seems no link to the appearance of wheeled transport in Ireland, at least based on current evidence.

### Construction of megalithic monuments—Enabling passage tomb architecture?

Ireland, like several northwest European regions in the 4th and 3rd millennia BC, is characterised by its megalithic architecture, and the link between megalith construction and the use of cattle for traction deserves consideration. In the Funnel Beaker (TRB) culture of northern Europe, evidence includes wheel tracks associated with the megalithic tomb at Flintbek [59, 60], engravings of cattle teams yoked to two-wheeled vehicles at the Züschen I megalithic tomb [61, 62], and the four-wheeled wagons with drawbars and yokes depicted on the pottery vessel from Bronocice [63]. In the later TRB, c. 3500 cal. BC onwards, it has been argued that land clearance for cultivation with the cattle-driven ard went hand in hand with the use of the retrieved material–mostly small and medium-sized glacial erratics—for megalith construction [64]. In 4th millennium BC Ireland, the picture is somewhat different and certainly more fragmented. As outlined above, based on the current state of knowledge, ard cultivation, wheeled transport and cattle traction seem not to appear simultaneously, and the size ranges of stones utilised in the construction of megalithic monuments frequently exceed those in TRB tombs.

Recent programmes of radiocarbon dating and mathematical modelling have also resulted in considerable blurring of traditional tomb typo-chronologies [65–69], with early passage tombs, court tombs and portal tombs all conceivably contemporary with one another and the Kilshane cattle. Nevertheless, the small amount of pottery from the Kilshane enclosure ditch, comprising a Middle Neolithic broad-rimmed globular bowl and a single sherd from a second globular bowl [22], links our traction data more closely to passage tomb horizons. The absence of evidence for cattle traction (and oxen) in the Irish Neolithic has created an understandable reluctance to speculate on the construction methods of passage tombs and megalithic monuments in general [70–74]. In the light of the Kilshane data, some well-recognised aspects of passage tombs as a monument class can be re-evaluated, namely their tendency to be sited at higher elevations than earlier monuments [75, 76] and with a high degree of inter-visibility, argued to reflect more extensively networked Middle Neolithic communities [75]. The earliest passage tomb activity recorded to date, at Carrowmore, Co. Sligo and Baltinglass, Co. Wicklow [67, 69], at c. 3700/3600 cal. BC, is in upland landscapes, with the Baltinglass tomb at an altitude of nearly 400 metres above sea level. So-called ‘developed’ passage tombs c. 3300–3000 cal. BC, such as those found 25 km to the north of Kilshane in the Boyne Valley, have long been recognised as incorporating kerbstones, orthostats and other stone elements sourced from long distances, up to 75 km in the case of quartz and granite cobbles from Newgrange [75, 77–80]. In these scenarios, cattle may have been used and even enabled the transport of both large and small stones over long distances and to higher terrain, as well as considerably easing efforts at a more local scale. Once on site, manoeuvring large structural stones into position would presumably have been easier with animal traction.

## Conclusion

The strong evidence of the exploitation of cattle for labour in the Middle Neolithic in Ireland fills a gap in our knowledge of the adoption of cattle traction in the northwest Atlantic islands and supports the revision of the Sherratt’s Secondary Products Revolution model by emphasizing the importance of local socio-economic contexts in the adoption of specific secondary products. The exploitation of draught cattle in Ireland appears to drive specialised herding practices producing large males, possibly oxen. Based on the current evidence, we argue that only a few selected individuals were used as draught animals. The acquisition of this technology has important implications for agriculture since it provides a key support for more extensive practices as well as for megalithic construction, which increases considerably in scale during this period. The presence of bones from draught cattle among the refuse of feasting events also raises the question of their status and whether the ownership of working animals was communal or in the hands of a privileged few. Regardless, it seems highly likely that access to draught animals and the exploitation of associated resources is at the heart of wider changes that took place in Neolithic society in the second half of the 4th millennium BC.

## Supporting information

S1 File (DOCX)
