# Supplementary material for: First evidence for cattle traction in Middle Neolithic Ireland: A pivotal element for resource exploitation
Source: PLoS One. 2023 Jan 26;18(1):e0279556. doi: 10.1371/journal.pone.0279556 (PMC9879418; doi:10.1371/journal.pone.0279556)
Supplement: S1 File — (DOCX) [file pone.0279556.s001.docx]

| Context | Sample No | Species | Element | Side | State of fusion | GL | GLl | Bp | SD | DD | Bd | BT | HTC | GLP | SLC | LA/LAR | M3 L | M3WA |
| --- | --- | --- | --- | --- | --- | --- | --- | --- | --- | --- | --- | --- | --- | --- | --- | --- | --- | --- |
| 5014 | 1562 | Cattle | Mandible | R |  | 0 | 0 | 0 | 0 | 0 | 0 | 0 | 0 | 0 | 0 | 0 | 37,1 | 16,2 |
| 5014 | 1562 | Cattle | Mandible | R |  | 0 | 0 | 0 | 0 | 0 | 0 | 0 | 0 | 0 | 0 | 0 | 37,1 | 16,1 |
| 5166 | 305 | Cattle | Mandible | L |  | 0 | 0 | 0 | 0 | 0 | 0 | 0 | 0 | 0 | 0 | 0 | 37,7 | 15,9 |
| 5166 | 314 | Cattle | Mandible | R |  | 0 | 0 | 0 | 0 | 0 | 0 | 0 | 0 | 0 | 0 | 0 | 0 | 15,9 |
| 5166 | 574 | Cattle | Mandible | L |  | 0 | 0 | 0 | 0 | 0 | 0 | 0 | 0 | 0 | 0 | 0 | 37,8 | 15,9 |
| 5164 | 1039 | Cattle | Radius | R | PF | 0 | 0 | 80,4 | 0 | 0 | 0 | 0 | 0 | 0 | 0 | 0 | 0 | 0 |
| 5164 | 1045 | Cattle | Astragalus | R |  | 0 | 60,5 | 0 | 0 | 0 | 42,3 | 0 | 0 | 0 | 0 | 0 | 0 | 0 |
| 5418 | 1062 | Cattle | Astragalus | R |  | 0 | 75,8 | 0 | 0 | 0 | 47,1 | 0 | 0 | 0 | 0 | 0 | 0 | 0 |
| 5418 | 1062 | Cattle | Scapula | L | PF | 0 | 0 | 0 | 0 | 0 | 0 | 0 | 0 | 65,5 | 0 | 0 | 0 | 0 |
| 5014 | 1100 | Cattle | Scapula | R | PF | 0 | 0 | 0 | 0 | 0 | 0 | 0 | 0 | 71,9 | 52,9 | 0 | 0 | 0 |
| 5014 | 1101 | Cattle | Scapula | L | PF | 0 | 0 | 0 | 0 | 0 | 0 | 0 | 0 | 71,2 | 50,1 | 0 | 0 | 0 |
| 5418 | 1112 | Cattle | Metacarpal | L | DF | 209,1 | 0 | 63,2 | 34,9 | 0 | 63,3 |  | 0 | 0 | 0 | 0 | 0 | 0 |
| 5014 | 1117 | Cattle | Astragalus | L |  | 0 | 70,5 | 0 | 0 | 0 | 44,1 | 0 | 0 | 0 | 0 | 0 | 0 | 0 |
| 5014 | 1118 | Cattle | Pelvis | L | PF | 0 | 0 | 0 | 0 | 0 | 0 | 0 | 0 | 0 | 0 | 55,4 | 0 | 0 |
| 5418 | 1119 | Cattle | Metacarpal | R | DF | 208,1 | 0 | 62,8 | 33,4 | 0 | 64,4 | 0 | 0 | 0 | 0 | 0 | 0 | 0 |
| 5418 | 1119 | Cattle | Radius | L | PF, DUX | 0 | 0 | 87,8 | 0 | 0 | 0 | 0 | 0 | 0 | 0 | 0 | 0 | 0 |
| 5418 | 1119 | Cattle | Astragalus | L |  | 0 | 66,3 | 0 | 0 | 0 | 47,9 | 0 | 0 | 0 | 0 | 0 | 0 | 0 |
| 5418 | 1119 | Cattle | Astragalus | R |  | 0 | 70,2 | 0 | 0 | 0 | 46,1 | 0 | 0 | 0 | 0 | 0 | 0 | 0 |
| 5418 | 1119 | Cattle | Tibia | R | PUM, DF | 0 | 0 | 0 | 0 | 0 | 63,2 | 0 | 0 | 0 | 0 | 0 | 0 | 0 |
| 5418 | 1119 | Cattle | Metatarsal | L | DF | 237,7 | 0 | 52,1 | 30,4 | 0 | 64,4 | 0 | 0 | 0 | 0 | 0 | 0 | 0 |
| 5418 | 1120 | Cattle | Scapula | L | PF | 0 | 0 | 0 | 0 | 0 | 0 | 0 | 0 | 69,7 | 54,1 | 0 | 0 | 0 |
| 5014 | 1121 | Cattle | Tibia | L | PUM, DFG | 0 | 0 | 0 | 0 | 0 | 92,5 | 0 | 0 | 0 | 0 | 0 | 0 | 0 |

Table S1. Measurements of cattle bones from the Kilshane enclosure site. PF, Proximal Fused, DF, Distal Fused, UX, unfused metaphysis & epiphysis; UM, unfused metaphysis. The abbreviations for the measurements are those provided by von den Driesch (1976).

| Context | Sample No | Species | Element | Side | State of fusion | GL | GLl | Bp | SD | DD | Bd | BT | HTC | GLP | SLC | LA/LAR | M3 L | M3WA |
| --- | --- | --- | --- | --- | --- | --- | --- | --- | --- | --- | --- | --- | --- | --- | --- | --- | --- | --- |
| 5418 | 1124 | Cattle | Metatarsal | L | DF | 243,2 | 0 | 51,5 | 31,3 | 0 | 61,7 | 0 | 0 | 0 | 0 | 0 | 0 | 0 |
| 5014 | 1130 | Cattle | Astragalus | R |  | 0 | 71,2 | 0 | 0 | 0 | 44,4 | 0 | 0 | 0 | 0 | 0 | 0 | 0 |
| 5014 | 1130 | Cattle | Radius | L | PF, DUX | 0 | 0 | 94,9 | 0 | 0 | 0 | 0 | 0 | 0 | 0 | 0 | 0 | 0 |
| 5525 | 1131 | Cattle | Tibia | R | DFG | 0 | 0 | 0 | 0 | 0 | 67,1 | 0 | 0 | 0 | 0 | 0 | 0 | 0 |
| 5525 | 1132 | Cattle | Humerus | L | PUX, DFG | 0 | 0 | 0 | 0 | 0 | 0 | 86,1 | 0 | 0 | 0 | 0 | 0 | 0 |
| 5014 | 1141 | Cattle | Metatarsal | R | DUX | 0 | 0 | 46,8 | 0 | 0 | 0 | 0 | 0 | 0 | 0 | 0 | 0 | 0 |
| 5014 | 1152 | Cattle | Astragalus | L |  | 0 | 56,3 | 0 | 0 | 0 | 43,8 | 0 | 0 | 0 | 0 | 0 | 0 | 0 |
| 5014 | 1153 | Cattle | Radius | L | PF | 0 | 0 | 82,2 | 0 | 0 | 0 | 0 | 0 | 0 | 0 | 0 | 0 | 0 |
| 5014 | 1153 | Cattle | Metacarpal | L | DUX | 0 | 0 | 60,1 | 0 | 0 | 0 | 0 | 0 | 0 | 0 | 0 | 0 | 0 |
| 5014 | 1153 | Cattle | Metacarpal | R | DUM | 0 | 0 | 58,9 | 0 | 0 | 0 | 0 | 0 | 0 | 0 | 0 | 0 | 0 |
| 5418 | 1204 | Cattle | Metacarpal | L | DF | 217,5 | 0 | 66,4 | 38,1 | 0 | 68,2 | 0 | 0 | 0 | 0 | 0 | 0 | 0 |
| 5418 | 1205 | Cattle | Metatarsal | R | DF | 242,2 | 0 | 52,4 | 30,2 | 0 | 67,4 | 0 | 0 | 0 | 0 | 0 | 0 | 0 |
| 5418 | 1208 | Cattle | Metacarpal | R | DF | 0 | 0 | 67,9 | 0 | 0 | 0 | 0 | 0 | 0 | 0 | 0 | 0 | 0 |
| 5418 | 1213 | Cattle | Metacarpal | L | DF | 211,5 | 0 | 67,1 | 39,9 | 0 | 69 | 0 | 0 | 0 | 0 | 0 | 0 | 0 |
| 5418 | 1214 | Cattle | Metacarpal | R | DF | 209,5 | 0 | 64,9 | 34,8 | 0 | 63,4 | 0 | 0 | 0 | 0 | 0 | 0 | 0 |
| 5418 | 1214 | Cattle | Metatarsal | R | DF | 248,3 | 0 | 51,8 | 31,1 | 0 | 62 | 0 | 0 | 0 | 0 | 0 | 0 | 0 |
| 1514 | 1216 | Cattle | Metacarpal | L | DF | 190 | 0 | 55,8 | 32,4 | 0 | 57,1 | 0 | 0 | 0 | 0 | 0 | 0 | 0 |
| 5014 | 1216 | Cattle | Metacarpal | R | DF | 188 | 0 | 57,4 | 30,5 | 0 | 59,4 | 0 | 0 | 0 | 0 | 0 | 0 | 0 |
| 5014 | 1216 | Cattle | Radius | L | PF, DUX | 0 | 0 | 77,5 | 0 | 0 | 0 | 0 | 0 | 0 | 0 | 0 | 0 | 0 |
| 5014 | 1216 | Cattle | Scapula | L | PF | 0 | 0 | 0 | 0 | 0 | 0 | 0 | 0 | 63,3 | 53,1 | 0 | 0 | 0 |
| 5418 | 1226 | Cattle | Metatarsal | R | DF | 245,1 | 0 | 50,3 | 30,3 | 0 | 58,7 | 0 | 0 | 0 | 0 | 0 | 0 | 0 |

Table S1. Measurements of cattle bones from the Kilshane enclosure site. PF, Proximal Fused, DF, Distal Fused, UX, unfused metaphysis & epiphysis; UM, unfused metaphysis. The abbreviations for the measurements are those provided by von den Driesch (1976).

| Context | Sample No | Species | Element | Side | State of fusion | GL | GLl | Bp | SD | DD | Bd | BT | HTC | GLP | SLC | LA/LAR | M3 L | M3WA |
| --- | --- | --- | --- | --- | --- | --- | --- | --- | --- | --- | --- | --- | --- | --- | --- | --- | --- | --- |
| 5014 | 1238 | Cattle | Tibia | R | PUX, DUX | 0 | 0 | 0 | 36,5 | 40,5 | 53,1 | 0 | 0 | 0 | 0 | 0 | 0 | 0 |
| 5014 | 1238 | Cattle | Astragalus | R |  | 0 | 57,9 | 0 | 0 | 0 | 38,6 | 0 | 0 | 0 | 0 | 0 | 0 | 0 |
| 5014 | 1238 | Cattle | Astragalus | L |  | 0 | 59,1 | 0 | 0 | 0 | 40,2 | 0 | 0 | 0 | 0 | 0 | 0 | 0 |
| 5014 | 1238 | Cattle | Metatarsal | R | DF | 215 | 0 | 42,6 | 25,1 | 0 | 53,1 | 0 | 0 | 0 | 0 | 0 | 0 | 0 |
| 5014 | 1238 | Cattle | Metatarsal | L | DF | 220 | 0 | 41,9 | 25,1 | 0 | 0 | 0 | 0 | 0 | 0 | 0 | 0 | 0 |
| 5014 | 1238 | Cattle | Tibia | L | PUX, DF | 0 | 0 | 0 | 37,1 | 41,1 | 54,9 | 0 | 0 | 0 | 0 | 0 | 0 | 0 |
| 5418 | 1240 | Cattle | Scapula | R | DF | 0 | 0 | 0 | 0 | 0 | 0 | 0 | 0 | 65 | 51,5 | 0 | 0 | 0 |
| 5418 | 1240 | Cattle | Astragalus | L |  | 0 | 70,6 | 0 | 0 | 0 | 44 | 0 | 0 | 0 | 0 | 0 | 0 | 0 |
| 5418 | 1240 | Cattle | Humerus | L | DF | 0 | 0 | 0 | 0 | 0 | 0 | 33,5 | 0 | 0 | 0 | 0 | 0 | 0 |
| 5418 | 1245 | Cattle | Metatarsal | U | DF | 236,1 | 0 | 49,2 | 28,2 | 0 | 55,9 | 0 | 0 | 0 | 0 | 0 | 0 | 0 |
| 5418 | 1262 | Cattle | Metacarpal | R | DF | 209,5 | 0 | 65,3 | 38,9 | 0 | 69,7 | 0 | 0 | 0 | 0 | 0 | 0 | 0 |
| 5418 | 1288 | Cattle | Metacarpal | L | DF | 200,1 | 0 | 61,1 | 35,3 | 0 | 63,6 | 0 | 0 | 0 | 0 | 0 | 0 | 0 |
| 5418 | 1303 | Cattle | Radius | L | PF, DUX | 0 | 0 | 73,5 | 0 | 0 | 0 | 0 | 0 | 0 | 0 | 0 | 0 | 0 |
| 5418 | 1303 | Cattle | Metacarpal | R | DF | 200,3 | 0 | 59,8 | 34,9 | 0 | 63,1 | 0 | 0 | 0 | 0 | 0 | 0 | 0 |
| 5418 | 1303 | Cattle | Astragalus | R |  | 0 | 63 | 0 | 0 | 0 | 41,8 | 0 | 0 | 0 | 0 | 0 | 0 | 0 |
| 5418 | 1303 | Cattle | Astragalus | L |  | 0 | 63 | 0 | 0 | 0 | 39,9 | 0 | 0 | 0 | 0 | 0 | 0 | 0 |
| 5791 | 1355 | Cattle | Scapula | L | DF | 0 | 0 | 0 | 0 | 0 | 0 | 0 | 0 | 73,5 | 0 | 0 | 0 | 0 |
| 5169 | 136 | Cattle | Astragalus | L |  | 0 | 66,8 | 0 | 0 | 0 | 43,1 | 0 | 0 | 0 | 0 | 0 | 0 | 0 |
| 5161 | 137 | Cattle | Radius | L | PF, DUX | 0 | 0 | 87,3 | 0 | 0 | 0 | 0 | 0 | 0 | 0 | 0 | 0 | 0 |

Table S1. Measurements of cattle bones from the Kilshane enclosure site. PF, Proximal Fused, DF, Distal Fused, UX, unfused metaphysis & epiphysis; UM, unfused metaphysis. The abbreviations for the measurements are those provided by von den Driesch (1976).

| Context | Sample No | Species | Element | Side | State of fusion | GL | GLl | Bp | SD | DD | Bd | BT | HTC | GLP | SLC | LA/LAR | M3 L | M3WA |
| --- | --- | --- | --- | --- | --- | --- | --- | --- | --- | --- | --- | --- | --- | --- | --- | --- | --- | --- |
| 5325 | 1381 | Cattle | Metacarpal | U | DF | 207,3 | 0 | 61,2 | 34,8 | 0 | 63 | 0 | 0 | 0 | 0 | 0 | 0 | 0 |
| 5325 | 1381 | Cattle | Radius | R | PF, DUX | 0 | 0 | 90,5 | 0 | 0 | 0 | 0 | 0 | 0 | 0 | 0 | 0 | 0 |
| 5538 | 1382 | Cattle | Astragalus | R |  | 0 | 59,1 | 0 | 0 | 0 | 45 | 0 | 0 | 0 | 0 | 0 | 0 | 0 |
| 5538 | 1382 | Cattle | Astragalus | L |  | 0 | 70 | 0 | 0 | 0 | 45,1 | 0 | 0 | 0 | 0 | 0 | 0 | 0 |
| 5325 | 1384 | Cattle | Astragalus | L |  | 0 | 66,1 | 0 | 0 | 0 | 42,7 | 0 | 0 | 0 | 0 | 0 | 0 | 0 |
| 5014 | 1388 | Cattle | Astragalus | R |  | 0 | 67,9 | 0 | 0 | 0 | 43,2 | 0 | 0 | 0 | 0 | 0 | 0 | 0 |
| 5014 | 1389 | Cattle | Astragalus | L |  | 0 | 65,1 | 0 | 0 | 0 | 40,5 | 0 | 0 | 0 | 0 | 0 | 0 | 0 |
| 5161 | 139 | Cattle | Metacarpal | R |  | 0 | 0 | 60,1 | 0 | 0 | 0 | 0 | 0 | 0 | 0 | 0 | 0 | 0 |
| 5538 | 1390 | Cattle | Radius | L | PF | 0 | 0 | 90 | 0 | 0 | 0 | 0 | 0 | 0 | 0 | 0 | 0 | 0 |
| 5538 | 1390 | Cattle | Radius | R | PF | 0 | 0 | 91,2 | 0 | 0 | 0 | 0 | 0 | 0 | 0 | 0 | 0 | 0 |
| 5538 | 1391 | Cattle | Humerus | L | PUX, DF | 0 | 0 | 0 | 0 | 0 | 0 | 78,9 | 35,9 | 0 | 0 | 0 | 0 | 0 |
| 5014 | 1393 | Cattle | Astragalus | R |  | 0 | 67,3 | 0 | 0 | 0 | 44,1 | 0 | 0 | 0 | 0 | 0 | 0 | 0 |
| 5538 | 1394 | Cattle | Scapula | L | DF | 0 | 0 | 0 | 0 | 0 | 0 | 0 | 0 | 73,4 |  | 0 | 0 | 0 |
| 5014 | 1398 | Cattle | Scapula | L | DF | 0 | 0 | 0 | 0 | 0 | 0 | 0 | 0 | 67,2 | 49,4 | 0 | 0 | 0 |
| 5014 | 1398 | Cattle | Astragalus | R |  | 0 | 65,5 | 0 | 0 | 0 | 40,6 | 0 | 0 | 0 | 0 | 0 | 0 | 0 |
| 5161 | 140 | Cattle | Pelvis | U | Acetabulum F | 0 | 0 | 0 | 0 | 0 | 0 | 0 | 0 | 0 | 0 | 67,8 | 0 | 0 |
| 5014 | 1402 | Cattle | Pelvis | R | Acetabulum F | 0 | 0 | 0 | 0 | 0 | 0 | 0 | 0 | 0 | 0 | 67,1 | 0 | 0 |
| 5380 | 1406 | Cattle | Radius | L | PF, DUM | 0 | 0 | 92,9 | 0 | 0 | 0 | 0 | 0 | 0 | 0 | 0 | 0 | 0 |
| 5380 | 1406 | Cattle | Astragalus | L |  | 0 | 65 | 0 | 0 | 0 | 44,4 | 0 | 0 | 0 | 0 | 0 | 0 | 0 |
| 5014 | 1409 | Cattle | Pelvis | L | Acetabulum F | 0 | 0 | 0 | 0 | 0 | 0 | 0 | 0 | 0 | 0 | 69 | 0 | 0 |
| 5380 | 1417 | Cattle | Radius | L | PF, DUM | 0 | 0 | 81,5 | 0 | 0 | 0 | 0 | 0 | 0 | 0 | 0 | 0 | 0 |

Table S1. Measurements of cattle bones from the Kilshane enclosure site. PF, Proximal Fused, DF, Distal Fused, UX, unfused metaphysis & epiphysis; UM, unfused metaphysis. The abbreviations for the measurements are those provided by von den Driesch (1976).

| Context | Sample No | Species | Element | Side | State of fusion | GL | GLl | Bp | SD | DD | Bd | BT | HTC | GLP | SLC | LA/LAR | M3 L | M3WA |
| --- | --- | --- | --- | --- | --- | --- | --- | --- | --- | --- | --- | --- | --- | --- | --- | --- | --- | --- |
| 5380 | 1417 | Cattle | Radius | R | PF, DUX | 0 | 0 | 84,2 | 0 | 0 | 0 | 0 | 0 | 0 | 0 | 0 | 0 | 0 |
| 5380 | 1417 | Cattle | Scapula | R | DF | 0 | 0 | 0 | 0 | 0 | 0 | 0 | 0 | 70,1 | 49,9 | 0 | 0 | 0 |
| 5380 | 1418 | Cattle | Metacarpal | R | PF, DUX | 0 | 0 | 63,3 | 0 | 0 | 0 | 0 | 0 | 0 | 0 | 0 | 0 | 0 |
| 5380 | 1418 | Cattle | Metacarpal | L | DUX | 0 | 0 | 60,9 | 0 | 0 |  | 0 | 0 | 0 | 0 | 0 | 0 | 0 |
| 5380 | 1418 | Cattle | Metacarpal | L | DUX | 0 | 0 | 61,1 | 0 | 0 | 0 | 0 | 0 | 0 | 0 | 0 | 0 | 0 |
| 5380 | 1418 | Cattle | Astragalus | L |  | 0 | 58,1 | 0 | 0 | 0 | 44,2 | 0 | 0 | 0 | 0 | 0 | 0 | 0 |
| 5380 | 1418 | Cattle | Astragalus | R |  | 0 | 67 | 0 | 0 | 0 | 44 | 0 | 0 | 0 | 0 | 0 | 0 | 0 |
| 5380 | 1418 | Cattle | Scapula | R | DF | 0 | 0 | 0 | 0 | 0 | 0 | 0 | 0 | 69,1 | 59,2 | 0 | 0 | 0 |
| 5380 | 1418 | Cattle | Astragalus | L |  | 0 | 72,9 | 0 | 0 | 0 | 45,2 | 0 | 0 | 0 | 0 | 0 | 0 | 0 |
| 5538 | 1429 | Cattle | Astragalus | R |  | 0 | 67,5 | 0 | 0 | 0 | 43,5 | 0 | 0 | 0 | 0 | 0 | 0 | 0 |
| 5538 | 1429 | Cattle | Astragalus | L |  | 0 | 68,1 | 0 | 0 | 0 | 41,2 | 0 | 0 | 0 | 0 | 0 | 0 | 0 |
| 5161 | 143 | Cattle | Scapula | L | DF | 0 | 0 | 0 | 0 | 0 | 0 | 0 | 0 | 71,4 | 0 | 0 | 0 | 0 |
| 5161 | 145 | Cattle | Humerus | R | PUM, DF | 0 | 0 | 0 | 0 | 0 | 0 | 83,1 | 34,6 | 0 | 0 | 0 | 0 | 0 |
| 5380 | 1458 | Cattle | Pelvis | R | Acetabulum F | 0 | 0 | 0 | 0 | 0 | 0 | 0 | 0 | 0 | 0 | 69,9 | 0 | 0 |
| 5169 | 146 | Cattle | Radius | R | DUX | 0 | 0 | 67,3 | 0 | 0 | 0 | 0 | 0 | 0 | 0 | 0 | 0 | 0 |
| 5380 | 1460 | Cattle | Tibia | R | PUM, DF | 0 | 0 | 0 | 0 | 0 | 62,7 | 0 | 0 | 0 | 0 | 0 | 0 | 0 |
| 5380 | 1460 | Cattle | Radius | L | PF, DUM | 0 | 0 | 88,2 | 0 | 0 | 0 | 0 | 0 | 0 | 0 | 0 | 0 | 0 |
| 5380 | 1467 | Cattle | Radius | L |  | 0 | 0 | 81,4 | 0 | 0 | 0 | 0 | 0 | 0 | 0 | 0 | 0 | 0 |
| 5169 | 147 | Cattle | Metatarsal | L | DF | 0 | 0 | 0 | 0 | 0 | 58,9 | 0 | 0 | 0 | 0 | 0 | 0 | 0 |
| 5169 | 147 | Cattle | Radius | L | PF, DUM | 0 | 0 | 80,4 | 0 | 0 | 0 | 0 | 0 | 0 | 0 | 0 | 0 | 0 |

Table S1. Measurements of cattle bones from the Kilshane enclosure site. PF, Proximal Fused, DF, Distal Fused, UX, unfused metaphysis & epiphysis; UM, unfused metaphysis. The abbreviations for the measurements are those provided by von den Driesch (1976).

| Context | Sample No | Species | Element | Side | State of fusion | GL | GLl | Bp | SD | DD | Bd | BT | HTC | GLP | SLC | LA/LAR | M3 L | M3WA |
| --- | --- | --- | --- | --- | --- | --- | --- | --- | --- | --- | --- | --- | --- | --- | --- | --- | --- | --- |
| 5014 | 1533 | Cattle | Astragalus | L |  | 0 | 63,3 | 0 | 0 | 0 | 42 | 0 | 0 | 0 | 0 | 0 | 0 | 0 |
| 5014 | 1552 | Cattle | Metatarsal | L | DF | 240 | 0 | 48,1 | 25,9 | 0 | 56,1 | 0 | 0 | 0 | 0 | 0 | 0 | 0 |
| 5014 | 1561 | Cattle | Metacarpal | L | DF | 196 | 0 | 57,4 | 32 | 0 | 0 | 0 | 0 | 0 | 0 | 0 | 0 | 0 |
| 5014 | 1561 | Cattle | Radius | L | PF | 0 | 0 | 93,1 | 0 | 0 | 0 | 0 | 0 | 0 | 0 | 0 | 0 | 0 |
| 5014 | 1561 | Cattle | Radius | L | PF, DUX | 0 | 0 | 76,5 | 0 | 0 | 0 | 0 | 0 | 0 | 0 | 0 | 0 | 0 |
| 5014 | 1561 | Cattle | Radius | R | PF, DUM | 0 | 0 | 84,9 | 0 | 0 | 0 | 0 | 0 | 0 | 0 | 0 | 0 | 0 |
| 5014 | 1561 | Cattle | Scapula | L | PFG | 0 | 0 | 0 | 0 | 0 | 0 | 0 | 0 | 61,3 | 45,1 | 0 | 0 | 0 |
| 5014 | 1566 | Cattle | Metacarpal | R |  | 0 | 0 | 56,5 | 0 | 0 | 0 | 0 | 0 | 0 | 0 | 0 | 0 | 0 |
| 5014 | 1566 | Cattle | Scapula | L | DF | 0 | 0 | 0 | 0 | 0 | 0 | 0 | 0 | 80,1 | 0 | 0 | 0 | 0 |
| 5014 | 1566 | Cattle | Tibia | L | PF, DF | 0 | 0 | 0 | 44,3 | 0 | 66 | 0 | 0 | 0 | 0 | 0 | 0 | 0 |
| 5014 | 1566 | Cattle | Radius | R | PF, DF | 338,8 | 0 | 99 | 48,9 | 0 | 65,7 | 0 | 0 | 0 | 0 | 0 | 0 | 0 |
| 5014 | 1566 | Cattle | Femur | R | DF | 0 | 0 | 0 | 0 | 0 | 105 | 0 | 0 | 0 | 0 | 0 | 0 | 0 |
| 5014 | 1566 | Cattle | Metacarpal | L | DF | 199,6 | 0 | 64,8 | 40,4 | 0 | 69,1 | 0 | 0 | 0 | 0 | 0 | 0 | 0 |
| 5014 | 1566 | Cattle | Calcaneum | R | PF | 146,9 | 0 | 0 | 0 | 0 | 0 | 0 | 0 | 0 | 0 | 0 | 0 | 0 |
| 5014 | 1567 | Cattle | Calcaneum | R | PF | 148,2 | 0 | 0 | 0 | 0 | 0 | 0 | 0 | 0 | 0 | 0 | 0 | 0 |
| 5014 | 1567 | Cattle | Astragalus | L |  | 0 | 70,3 | 0 | 0 | 0 | 47,2 | 0 | 0 | 0 | 0 | 0 | 0 | 0 |
| 5014 | 1567 | Cattle | Astragalus | R |  | 0 | 70,1 | 0 | 0 | 0 | 48,5 | 0 | 0 | 0 | 0 | 0 | 0 | 0 |
| 5014 | 1567 | Cattle | Metatarsal | R | DF | 237,7 | 0 | 51,9 | 30,4 | 0 | 63,9 | 0 | 0 | 0 | 0 | 0 | 0 | 0 |
| 5014 | 1567 | Cattle | Metacarpal | R | DF | 205,9 | 0 | 67,6 | 36,9 | 0 | 69,4 | 0 | 0 | 0 | 0 | 0 | 0 | 0 |
| 5014 | 1567 | Cattle | Humerus | L | PUM, DF | 0 | 0 | 0 | 0 | 0 | 0 | 78,3 | 34,9 | 0 | 0 | 0 | 0 | 0 |

Table S1. Measurements of cattle bones from the Kilshane enclosure site. PF, Proximal Fused, DF, Distal Fused, UX, unfused metaphysis & epiphysis; UM, unfused metaphysis. The abbreviations for the measurements are those provided by von den Driesch (1976).

| Context | Sample No | Species | Element | Side | State of fusion | GL | GLl | Bp | SD | DD | Bd | BT | HTC | GLP | SLC | LA/LAR | M3 L | M3WA |
| --- | --- | --- | --- | --- | --- | --- | --- | --- | --- | --- | --- | --- | --- | --- | --- | --- | --- | --- |
| 5014 | 1567 | Cattle | Metatarsal | L | DUX | 239,1 | 0 | 53,2 | 31,4 | 0 | 64,1 | 0 | 0 | 0 | 0 | 0 | 0 | 0 |
| 5014 | 1567 | Cattle | Astragalus | R |  | 0 | 70 | 0 | 0 | 0 | 44 | 0 | 0 | 0 | 0 | 0 | 0 | 0 |
| 5014 | 1567 | Cattle | Humerus | R | PUX, DF | 0 | 0 | 0 | 0 | 0 | 0 | 78,1 | 33,4 | 0 | 0 | 0 | 0 | 0 |
| 5014 | 1567 | Cattle | Astragalus | R |  | 0 | 70,5 | 0 | 0 | 0 | 44,8 | 0 | 0 | 0 | 0 | 0 | 0 | 0 |
| 5014 | 1567 | Cattle | Tibia | L | DF | 0 | 0 | 0 | 0 | 0 | 59,1 | 0 | 0 | 0 | 0 | 0 | 0 | 0 |
| 5014 | 1567 | Cattle | Humerus | R | PF, DF | 0 | 0 | 0 | 0 | 0 | 0 | 87,8 | 38,2 | 0 | 0 | 0 | 0 | 0 |
| 5014 | 1567 | Cattle | Scapula | R | DF | 0 | 0 | 0 | 0 | 0 | 0 | 0 | 0 | 82,3 | 58,9 | 0 | 0 | 0 |
| 5014 | 1568 | Cattle | Radius | R | PF | 0 | 0 | 86,9 | 0 | 0 | 0 | 0 | 0 | 0 | 0 | 0 | 0 | 0 |
| 5014 | 1568 | Cattle | Tibia | R | PF, DF | 395 | 0 | 107 | 45,1 | 0 | 69,5 | 0 | 0 | 0 | 0 | 0 | 0 | 0 |
| 5014 | 1568 | Cattle | Calcaneum | R | F | 63,9 | 0 | 0 | 0 | 0 | 0 | 0 | 0 | 0 | 0 | 0 | 0 | 0 |
| 5014 | 1568 | Cattle | Astragalus | L |  | 0 | 70,2 | 0 | 0 | 0 | 46,1 | 0 | 0 | 0 | 0 | 0 | 0 | 0 |
| 5014 | 1568 | Cattle | Humerus | L | PUX, DF | 0 | 0 | 0 | 0 | 0 | 0 | 79,4 | 34,8 | 0 | 0 | 0 | 0 | 0 |
| 5014 | 1568 | Sheep | Humerus | L | DF | 0 | 0 | 0 | 0 | 0 | 0 | 25,4 | 14,8 | 0 | 0 | 0 | 0 | 0 |
| 5014 | 1568 | Cattle | Radius | L | PF, DUX | 0 | 0 | 86 | 0 | 0 | 0 | 0 | 0 | 0 | 0 | 0 | 0 | 0 |
| 5014 | 1571 | Cattle | Calcaneum | L | PF | 149,5 | 0 | 0 | 0 | 0 | 0 | 0 | 0 | 0 | 0 | 0 | 0 | 0 |
| 5014 | 1572 | Cattle | Metatarsal | R | DF | 240 | 0 | 47,9 | 26,9 | 0 | 57,1 |  | 0 | 0 | 0 | 0 | 0 | 0 |
| 5014 | 1572 | Cattle | Radius | R | PF, DUM | 0 | 0 | 87,1 | 0 | 0 | 0 | 0 | 0 | 0 | 0 | 0 | 0 | 0 |
| 5014 | 1574 | Cattle | Metacarpal | R | DF | 206,9 | 0 | 62,9 | 33,3 | 0 | 64 | 0 | 0 | 0 | 0 | 0 | 0 | 0 |
| 5014 | 1574 | Cattle | Metacarpal | L | DF | 205 | 0 | 61,9 | 33,7 | 0 | 64,5 | 0 | 0 | 0 | 0 | 0 | 0 | 0 |

Table S1. Measurements of cattle bones from the Kilshane enclosure site. PF, Proximal Fused, DF, Distal Fused, UX, unfused metaphysis & epiphysis; UM, unfused metaphysis. The abbreviations for the measurements are those provided by von den Driesch (1976).

| Context | Sample No | Species | Element | Side | State of fusion | GL | GLl | Bp | SD | DD | Bd | BT | HTC | GLP | SLC | LA/LAR | M3 L | M3WA |
| --- | --- | --- | --- | --- | --- | --- | --- | --- | --- | --- | --- | --- | --- | --- | --- | --- | --- | --- |
| 5014 | 1574 | Sheep/ goat | Metacarpal | L |  | 0 | 0 | 21,1 | 12,4 | 0 | 0 | 0 | 0 | 0 | 0 | 0 | 0 | 0 |
| 5014 | 1577 | Cattle | Metacarpal | R | DUX | 0 | 0 | 63,6 | 0 | 0 | 0 | 0 | 0 | 0 | 0 | 0 | 0 | 0 |
| 5164 | 190 | Cattle | Astragalus | R |  | 0 | 74,1 | 0 | 0 | 0 | 46,9 | 0 | 0 | 0 | 0 | 0 | 0 | 0 |
| 5164 | 190 | Cattle | Radius | R | PF, DUM | 0 | 0 | 83,9 | 0 | 0 | 0 | 0 | 0 | 0 | 0 | 0 | 0 | 0 |
| 5164 | 229 | Cattle | Scapula | R | DF | 0 | 0 | 0 | 0 | 0 | 0 | 0 | 0 | 0 | 52,3 | 0 | 0 | 0 |
| 5164 | 239 | Cattle | Astragalus | L |  | 0 | 67,1 | 0 | 0 | 0 | 43,1 | 0 | 0 | 0 | 0 | 0 | 0 | 0 |
| 5164 | 239 | Cattle | Astragalus | R |  | 0 | 66,1 | 0 | 0 | 0 | 42,9 | 0 | 0 | 0 | 0 | 0 | 0 | 0 |
| 5164 | 239 | Cattle | Astragalus | R |  | 0 | 68,3 | 0 | 0 | 0 | 40,3 | 0 | 0 | 0 | 0 | 0 | 0 | 0 |
| 5164 | 252 | Cattle | Astragalus | R |  | 0 | 68,1 | 0 | 0 | 0 | 41,9 | 0 | 0 | 0 | 0 | 0 | 0 | 0 |
| 5164 | 279 | Cattle | Scapula | L |  | 0 | 0 | 0 | 0 | 0 | 0 | 0 | 0 | 68,1 | 50,9 | 0 | 0 | 0 |
| 5164 | 281 | Cattle | Scapula | R | PF | 0 | 0 | 0 | 0 | 0 | 0 | 0 | 0 | 68 | 0 | 0 | 0 | 0 |
| 5164 | 281 | Cattle | Humerus | R | DFG | 0 | 0 | 0 | 0 | 0 | 0 | 77,9 | 33,1 | 0 | 0 | 0 | 0 | 0 |
| 5166 | 306 | Cattle | Metatarsal | R | DUM | 0 | 0 | 50,9 | 0 | 0 | 0 | 0 | 0 | 0 | 0 | 0 | 0 | 0 |
| 5166 | 306 | Cattle | Tibia | R | DFG | 0 | 0 | 0 | 0 | 0 | 63,9 | 0 | 0 | 0 | 0 | 0 | 0 | 0 |
| 5164 | 329 | Cattle | Radius | R | PF, DUM | 0 | 0 | 85,5 | 0 | 0 | 0 | 0 | 0 | 0 | 0 | 0 | 0 | 0 |
| 5164 | 329 | Cattle | Radius | L | PF, DUM | 0 | 0 | 84,1 | 0 | 0 | 0 | 0 | 0 | 0 | 0 | 0 | 0 | 0 |
| 5164 | 329 | Cattle | Scapula | L | DF | 0 | 0 | 0 | 0 | 0 | 0 | 0 | 0 | 0 | 57,1 | 0 | 0 | 0 |
| 5164 | 329 | Cattle | Humerus | R | DF | 0 | 0 | 0 | 0 | 0 | 0 | 73,5 | 33,1 | 0 | 0 | 0 | 0 | 0 |
| 5014 | 390 | Cattle | Radius | L | PF, DUM | 0 | 0 | 81,9 | 0 | 0 | 0 | 0 | 0 | 0 | 0 | 0 | 0 | 0 |

Table S1. Measurements of cattle bones from the Kilshane enclosure site. PF, Proximal Fused, DF, Distal Fused, UX, unfused metaphysis & epiphysis; UM, unfused metaphysis. The abbreviations for the measurements are those provided by von den Driesch (1976).

| Context | Sample No | Species | Element | Side | State of fusion | GL | GLl | Bp | SD | DD | Bd | BT | HTC | GLP | SLC | LA/LAR | M3 L | M3WA |
| --- | --- | --- | --- | --- | --- | --- | --- | --- | --- | --- | --- | --- | --- | --- | --- | --- | --- | --- |
| 5014 | 392 | Cattle | Astragalus | L |  | 0 | 60 | 0 | 0 | 0 | 40,6 | 0 | 0 | 0 | 0 | 0 | 0 | 0 |
| 5166 | 397 | Cattle | Metacarpal | L |  | 0 | 0 | 60,5 | 0 | 0 | 0 | 0 | 0 | 0 | 0 | 0 | 0 | 0 |
| 5166 | 398 | Cattle | Metatarsal | L |  | 0 | 0 | 47,2 | 0 | 0 | 0 | 0 | 0 | 0 | 0 | 0 | 0 | 0 |
| 5166 | 398 | Cattle | Astragalus | L |  | 0 | 64,1 | 0 | 0 | 0 | 42,9 | 0 | 0 | 0 | 0 | 0 | 0 | 0 |
| 5166 | 398 | Cattle | Metatarsal | R |  | 0 | 0 | 45,9 | 0 | 0 | 0 | 0 | 0 | 0 | 0 | 0 | 0 | 0 |
| 5503 | 450 | Cattle | Astragalus | R |  | 0 | 66,9 | 0 | 0 | 0 | 46,1 | 0 | 0 | 0 | 0 | 0 | 0 | 0 |
| 5503 | 451 | Cattle | Metacarpal | L | DF | 196,5 | 0 | 58,2 | 35,8 | 0 | 62,2 | 0 | 0 | 0 | 0 | 0 | 0 | 0 |
| 5503 | 467 | Cattle | Metatarsal | R | PF, DF | 228,7 | 0 | 48,2 | 28,1 | 0 | 57,6 | 0 | 0 | 0 | 0 | 0 | 0 | 0 |
| 5418 | 493 | Cattle | Radius | R | PF, DUX | 0 | 0 | 89,2 | 0 | 0 | 0 | 0 | 0 | 0 | 0 | 0 | 0 | 0 |
| 5418 | 500 | Cattle | Humerus | R | PUX, DF | 0 | 0 | 0 | 0 | 0 | 0 | 79 | 34,1 | 0 | 0 | 0 | 0 | 0 |
| 5418B | 503 | Cattle | Radius | L | PF, DUM | 0 | 0 | 64,1 | 0 | 0 | 0 | 0 | 0 | 0 | 0 | 0 | 0 | 0 |
| 5418B | 504 | Cattle | Metatarsal | U | DF | 243 | 0 | 48,6 | 38,1 | 0 | 57,4 | 0 | 0 | 0 | 0 | 0 | 0 | 0 |
| 5418B | 505 | Cattle | Astragalus | L |  | 0 | 70,2 | 0 | 0 | 0 | 45,3 | 0 | 0 | 0 | 0 | 0 | 0 | 0 |
| 5418B | 505 | Cattle | Metatarsal | R | DF | 341,3 | 0 | 53,8 | 33,1 | 0 | 59,7 | 0 | 0 | 0 | 0 | 0 | 0 | 0 |
| 5538 | 511 | Cattle | Astragalus | L |  | 0 | 65,4 | 0 | 0 | 0 | 43,2 | 0 | 0 | 0 | 0 | 0 | 0 | 0 |
| 5538 | 511 | Cattle | Astragalus | L |  | 0 | 63,4 | 0 | 0 | 0 | 43,9 | 0 | 0 | 0 | 0 | 0 | 0 | 0 |
| 5538 | 511 | Cattle | Humerus | L | DF | 0 | 0 | 0 | 0 | 0 | 0 | 79,9 | 33,7 | 0 | 0 | 0 | 0 | 0 |
| 5538 | 520 | Cattle | Astragalus | R |  | 0 | 61,9 | 0 | 0 | 0 | 42,6 | 0 | 0 | 0 | 0 | 0 | 0 | 0 |
| 5538 | 522 | Cattle | Metacarpal | R | DF | 216,8 | 0 | 61,9 | 34,5 | 0 | 63 | 0 | 0 | 0 | 0 | 0 | 0 | 0 |

Table S1. Measurements of cattle bones from the Kilshane enclosure site. PF, Proximal Fused, DF, Distal Fused, UX, unfused metaphysis & epiphysis; UM, unfused metaphysis. The abbreviations for the measurements are those provided by von den Driesch (1976).

| Context | Sample No | Species | Element | Side | State of fusion | GL | GLl | Bp | SD | DD | Bd | BT | HTC | GLP | SLC | LA/LAR | M3 L | M3WA |
| --- | --- | --- | --- | --- | --- | --- | --- | --- | --- | --- | --- | --- | --- | --- | --- | --- | --- | --- |
| 5538 | 530 | Cattle | Radius | L | PF | 0 | 0 | 81,9 | 0 | 0 | 0 | 0 | 0 | 0 | 0 | 0 | 0 | 0 |
| 5538 | 532 | Cattle | Humerus | R | DF | 0 | 0 | 0 | 0 | 0 | 0 | 0 | 34,5 | 0 | 0 | 0 | 0 | 0 |
| 5538 | 535 | Cattle | Metacarpal | L | DF | 0 | 0 | 60,2 | 0 | 0 | 61,9 | 0 | 0 | 0 | 0 | 0 | 0 | 0 |
| 5380 | 554 | Cattle | Astragalus | L |  | 0 | 69,7 | 0 | 0 | 0 | 43 | 0 | 0 | 0 | 0 | 0 | 0 | 0 |
| 5380 | 554 | Cattle | Metacarpal | L | DF | 209,5 | 0 | 61,1 | 32,9 | 0 | 65,6 | 0 | 0 | 0 | 0 | 0 | 0 | 0 |
| 5380 | 557 | Cattle | Metacarpal | R | DUX | 0 | 0 | 60,6 | 0 | 0 | 0 | 0 | 0 | 0 | 0 | 0 | 0 | 0 |
| 5380 | 557 | Cattle | Humerus | R | PUX, PUM | 0 | 0 | 0 | 0 | 0 | 0 | 75,8 | 36,4 | 0 | 0 | 0 | 0 | 0 |
| 5380 | 559 | Cattle | Astragalus | L |  | 0 | 67,9 | 0 | 0 | 0 | 43,1 | 0 | 0 | 0 | 0 | 0 | 0 | 0 |
| 5380 | 560 | Sheep/ goat | Tibia | L | DUX | 0 | 0 | 0 | 0 | 0 | 24,5 | 0 | 0 | 0 | 0 | 0 | 0 | 0 |
| 5380 | 566 | Cattle | Metacarpal | R |  | 0 | 0 | 58,8 | 0 | 0 | 0 | 0 | 0 | 0 | 0 | 0 | 0 | 0 |
| 5166 | 576 | Cattle | Radius | L | PF, DUM | 0 | 0 | 81,4 | 0 | 0 | 0 | 0 | 0 | 0 | 0 | 0 | 0 | 0 |
| 5166 | 582 | Cattle | Astragalus | L |  | 0 | 65,9 | 0 | 0 | 0 | 42,2 | 0 | 0 | 0 | 0 | 0 | 0 | 0 |
| 5166 | 584 | Cattle | Metacarpal | L | PF, DUX | 0 | 0 | 60,1 | 0 | 0 | 0 | 0 | 0 | 0 | 0 | 0 | 0 | 0 |
| 5325 | 632 | Cattle | Scapula | R | DF | 0 | 0 | 0 | 0 | 0 | 0 | 0 | 0 | 69,6 | 0 | 0 | 0 | 0 |
| 5325 | 658 | Cattle | Humerus | L | DF | 0 | 0 | 0 | 0 | 0 | 0 | 0 | 35,5 | 0 | 0 | 0 | 0 | 0 |
| 5325 | 658 | Cattle | Pelvis | R | Acetabulum F | 0 | 0 | 0 | 0 | 0 | 0 | 0 | 0 | 0 | 0 | 67,3 | 0 | 0 |
| 5325 | 692 | Cattle | Radius | L | PF, DUX | 0 | 0 | 0 | 0 | 0 | 87,8 | 0 | 0 | 0 | 0 | 0 | 0 | 0 |
| 5325 | 694 | Cattle | Pelvis | L | Acetabulum F | 0 | 0 | 0 | 0 | 0 | 0 | 0 | 0 | 0 | 0 | 67,1 | 0 | 0 |
| 5325 | 705 | Cattle | Calcaneum | L | PF | 134,4 | 0 | 0 | 0 | 0 | 0 | 0 | 0 | 0 | 0 | 0 | 0 | 0 |

Table S1. Measurements of cattle bones from the Kilshane enclosure site. PF, Proximal Fused, DF, Distal Fused, UX, unfused metaphysis & epiphysis; UM, unfused metaphysis. The abbreviations for the measurements are those provided by von den Driesch (1976).

| Context | Sample No | Species | Element | Side | State of fusion | GL | GLl | Bp | SD | DD | Bd | BT | HTC | GLP | SLC | LA/LAR | M3 L | M3WA |
| --- | --- | --- | --- | --- | --- | --- | --- | --- | --- | --- | --- | --- | --- | --- | --- | --- | --- | --- |
| 5325 | 705 | Cattle | Calcaneum | R | PF | 133,8 | 0 | 0 | 0 | 0 | 0 | 0 | 0 | 0 | 0 | 0 | 0 | 0 |
| 5325 | 705 | Cattle | Tibia | R |  | 352 | 0 | 0 | 38,1 | 0 | 59,1 | 0 | 0 | 0 | 0 | 0 | 0 | 0 |
| 5325 | 705 | Cattle | Metatarsal | L | DF | 227,4 | 0 | 45 | 27,2 | 0 | 55,4 | 0 | 0 | 0 | 0 | 0 | 0 | 0 |
| 5325 | 723 | Cattle | Metatarsal | R | PF, DUX | 0 | 0 | 52,2 | 0 | 0 | 0 | 0 | 0 | 0 | 0 | 0 | 0 | 0 |
| 5325 | 724 | Cattle | Astragalus | R |  | 0 | 71,9 | 0 | 0 | 0 | 41,9 | 0 | 0 | 0 | 0 | 0 | 0 | 0 |
| 5325 | 724 | Cattle | Metacarpal | L | DUX | 0 | 0 | 64 | 0 | 0 | 0 | 0 | 0 | 0 | 0 | 0 | 0 | 0 |
| 5325 | 724 | Cattle | Radius | R | PF | 0 | 0 | 85,7 | 0 | 0 | 0 | 0 | 0 | 0 | 0 | 0 | 0 | 0 |
| 5483 | 737 | Pig | Tibia | R | DF | 0 | 0 | 0 | 0 | 0 | 37,9 | 0 | 0 | 0 | 0 | 0 | 0 | 0 |
| 5325 | 765 | Cattle | Tibia | L | DF | 0 | 0 | 0 | 0 | 0 | 62,8 | 0 | 0 | 0 | 0 | 0 | 0 | 0 |
| 5325 | 765 | Cattle | Astragalus | R |  | 0 | 65,2 | 0 | 0 | 0 | 43,2 | 0 | 0 | 0 | 0 | 0 | 0 | 0 |
| 5325 | 769 | Cattle | Metacarpal | L | DUX | 0 | 0 | 58,9 | 0 | 0 | 0 | 0 | 0 | 0 | 0 | 0 | 0 | 0 |
| 2325 | 770 | Cattle | Radius | R | DUX | 0 | 0 | 83,9 | 39,8 | 0 | 0 | 0 | 0 | 0 | 0 | 0 | 0 | 0 |
| 5325 | 774 | Cattle | Pelvis | R | Acetabulum F | 0 | 0 | 0 | 0 | 0 | 0 | 0 | 0 | 0 | 0 | 67,2 | 0 | 0 |
| 5325 | 774 | Cattle | Humerus | L | PF, DF | 0 | 0 | 0 | 0 | 0 | 0 | 0 | 34,9 | 0 | 0 | 0 | 0 | 0 |
| 5325 | 774 | Cattle | Radius | R | PF, DF | 289,5 | 0 | 82,4 | 41,4 | 0 | 72,4 | 0 | 0 | 0 | 0 | 0 | 0 | 0 |
| 5325 | 774 | Cattle | Radius | L | PF, DF | 291,4 | 0 | 82,1 | 40,1 | 0 | 71,9 | 0 | 0 | 0 | 0 | 0 | 0 | 0 |
| 5325 | 774 | Cattle | Metacarpal | L | DF | 199,2 | 0 | 0 | 31,1 | 0 | 59,9 | 0 | 0 | 0 | 0 | 0 | 0 | 0 |
| 5325 | 789 | Cattle | Metacarpal | L | DUX | 0 | 0 | 0 | 0 | 0 | 62,2 | 0 | 0 | 0 | 0 | 0 | 0 | 0 |
| 5325 | 803 | Cattle | Radius | R | PF, DUM | 0 | 0 | 86,8 | 0 | 0 | 0 | 0 | 0 | 0 | 0 | 0 | 0 | 0 |
| 5325 | 803 | Cattle | Astragalus | L |  | 0 | 72 | 0 | 0 | 0 | 42,9 | 0 | 0 | 0 | 0 | 0 | 0 | 0 |

Table S1. Measurements of cattle bones from the Kilshane enclosure site. PF, Proximal Fused, DF, Distal Fused, UX, unfused metaphysis & epiphysis; UM, unfused metaphysis. The abbreviations for the measurements are those provided by von den Driesch (1976).

| Context | Sample No | Species | Element | Side | State of fusion | GL | GLl | Bp | SD | DD | Bd | BT | HTC | GLP | SLC | LA/LAR | M3 L | M3WA |
| --- | --- | --- | --- | --- | --- | --- | --- | --- | --- | --- | --- | --- | --- | --- | --- | --- | --- | --- |
| 5325 | 804 | Cattle | Astragalus | L |  | 0 | 66,9 | 0 | 0 | 0 | 43,5 | 0 | 0 | 0 | 0 | 0 | 0 | 0 |
| 5325 | 805 | Cattle | Astragalus | R |  | 0 | 61,5 | 0 | 0 | 0 | 43,1 | 0 | 0 | 0 | 0 | 0 | 0 | 0 |
| 5325 | 806 | Cattle | Radius | R | PF, DUX | 0 | 0 | 87,2 | 0 | 0 | 0 | 0 | 0 | 0 | 0 | 0 | 0 | 0 |
| 5164 | 808 | Cattle | Astragalus | L |  | 0 | 65,6 | 0 | 0 | 0 | 42,5 | 0 | 0 | 0 | 0 | 0 | 0 | 0 |
| 5325 | 809 | Cattle | Metatarsal | R | DF | 230,1 | 0 | 47,2 | 27,1 | 0 | 65,2 | 0 | 0 | 0 | 0 | 0 | 0 | 0 |
| 5238 | 813 | Cattle | Tibia | L | DF | 0 | 0 | 0 | 0 | 0 | 63 | 0 | 0 | 0 | 0 | 0 | 0 | 0 |
| 5418 | 856 | Cattle | Pelvis | R | Acetabulum F | 0 | 0 | 0 | 0 | 0 | 0 | 0 | 0 | 0 | 0 | 77,3 | 0 | 0 |
| 5014 | 859 | Cattle | Astragalus | L |  | 0 | 67,3 | 0 | 0 | 0 | 43,9 | 0 | 0 | 0 | 0 | 0 | 0 | 0 |
| 5014 | 863 | Cattle | Astragalus | R |  | 0 | 67,5 | 0 | 0 | 0 | 42,5 | 0 | 0 | 0 | 0 | 0 | 0 | 0 |
| 5014 | 868 | Cattle | Pelvis | R | Acetabulum F | 0 | 0 | 0 | 0 | 0 | 0 | 0 | 0 | 0 | 0 | 71,9 | 0 | 0 |
| 5014 | 884 | Cattle | Astragalus | R |  | 0 | 64,1 | 0 | 0 | 0 | 45,6 | 0 | 0 | 0 | 0 | 0 | 0 | 0 |
| 5503 | 916 | Cattle | Tibia | L | PUX, DF | 0 | 0 | 0 | 0 | 0 | 61 | 0 | 0 | 0 | 0 | 0 | 0 | 0 |
| 5503 | 916 | Cattle | Tibia | R | DF | 0 | 0 | 0 | 0 | 0 | 65,1 | 0 | 0 | 0 | 0 | 0 | 0 | 0 |
| 5503 | 916 | Cattle | Metatarsal | L | DF | 229 | 0 | 47,1 | 28,8 | 0 | 58 | 0 | 0 | 0 | 0 | 0 | 0 | 0 |
| 5503 | 916 | Cattle | Humerus | R | PUX, DF | 0 | 0 | 0 | 0 | 0 | 0 | 80 | 32,1 | 0 | 0 | 0 | 0 | 0 |
| 5503 | 916 | Cattle | Astragalus | R |  | 0 | 69,7 | 0 | 0 | 0 | 44,6 | 0 | 0 | 0 | 0 | 0 | 0 | 0 |
| 5503 | 918 | Cattle | Metacarpal | R | DF | 198,5 | 0 | 57,9 | 34,4 | 0 | 61,3 | 0 | 0 | 0 | 0 | 0 | 0 | 0 |
| 5503 | 918 | Cattle | Humerus | L | PUX, DF | 0 | 0 | 0 | 0 | 0 | 0 | 78,8 | 33,9 | 0 | 0 | 0 | 0 | 0 |
| 5503 | 918 | Cattle | Radius | R | PF, DUX | 0 | 0 | 83,3 | 0 | 0 | 0 | 0 | 0 | 0 | 0 | 0 | 0 | 0 |
| 5503 | 918 | Cattle | Radius | L | PF, DUX | 0 | 0 | 86,4 | 0 | 0 | 0 | 0 | 0 | 0 | 0 | 0 | 0 | 0 |
| 5418 | 958 | Cattle | Scapula | L | DF | 0 | 0 | 0 | 0 | 0 | 0 | 0 | 0 | 80 | 57,8 | 0 | 0 | 0 |

Table S1. Measurements of cattle bones from the Kilshane enclosure site. PF, Proximal Fused, DF, Distal Fused, UX, unfused metaphysis & epiphysis; UM, unfused metaphysis. The abbreviations for the measurements are those provided by von den Driesch (1976).

| Context | Sample No | Element | Fusion | A/P | L/M | GL | Bp | SC | Bd | Pex | Plip | Dex | Prox osteo. | Dist osteo. | Comment |
| --- | --- | --- | --- | --- | --- | --- | --- | --- | --- | --- | --- | --- | --- | --- | --- |
| 1231 | 5001 | Ph1 | PF | A | L | 54,3 | 26,9 | 21,3 | 24,8 | 1 | 1 | 1 | 1 | 1 |  |
| 5014 | 1141 | Ph1 | PF | A | L | 64,1 | 29,5 | 24,7 | 27,8 | 2/1 | 1 | 1 | 1 | 1 | eroded |
| 5325 | 804 | Ph1 | PF | A | L | 61,3 | 32,5 | 26,6 | 29,9 | 1 | 1 | - | - | - | eroded |
| 5014 | 381 | Ph1 | PF | P | L | 62,9 | 28,1 | 23,1 | 25,7 | 1 | 1 | 2 | 1 | 1 |  |
| 5164 | 229 | Ph1 | PF | A | M | 61,9 | 31,6 | 25,3 | 29,2 | 1 | - | 2 | 1 | - |  |
| 5014 | 392 | Ph1 | PF | P | L | 59 | 30,7 | 24,9 | 28,5 | - | - | - | - | - | eroded |
| 5014 | 392 | Ph1 | PF | A | L | 60,1 | 27,6 | 20,7 | 26,1 | 2 | 1 | 2 | 1 | 1 |  |
| 5014 | 392 | Ph1 | PF | - | - | 59 | - | - | - | - | - | - | - | - | eroded |
| 5325 | 790 | Ph1 | PF | P | L | 63,1 | 27,1 | 21,6 | 25,3 | - | - | - | - | - | eroded |
| 5325 | 790 | Ph1 | PF | P | M | 61,8 | 28,1 | 23,8 | 27,4 | - | - | - | - | - | eroded |
| 5325 | 790 | Ph1 | PF | P | M | 62,9 | 28,6 | 23,6 | 27,5 | - | - | - | - | - | eroded |
| 5325 | 790 | Ph1 | PF | P | M | 62,7 | 27,2 | 22,1 | 25,9 | - | - | - | - | - | eroded |
| F 5418 | 1214 | Ph1 | PF | A | L | 64,4 | 31,9 | 26,8 | 31,3 | 2 | 2 | 2 | 2 | 1 | Same individual |
| F 5418 | 1214 | Ph2 | PF | A | L | 42,5 | 31,2 | 23,8 | 24 | 2 | 2 | 2 | 2 | 1 |  |
| 5538 | 521 | Ph1 | PF | P | M | 63,6 | 31,1 | 26,8 | 32,1 | 2 | 2 | 2 | 1 | 1 |  |
| 5014 | 1572 | Ph1 | PF | P | M | 63,2 | 30,1 | 22,8 | 28,5 | 1 | 1 | 2 | 1 | - | eroded |
| 5014 | 1572 | Ph2 | PF | P | - | 40,8 | 28,3 | 22,7 | 24,2 | 1 | 2 | 1 | - | - | eroded |
| 5014 | 1572 | Ph2 | PF | P | - | 41,3 | 30,6 | 23,3 | 24,4 | 2 | 2 | 1 | 1 | - | eroded |
| 5014 | 1572 | Ph1 | PF | P | - | 64,4 | 29,6 | 26,4 | 30,1 | 1/2 | 2 | 2 | 1 | - | eroded |
| 5014 | 1561 | Ph1 | PF | P | L | 63,9 | 32,4 | 25,5 | 29,4 | 2 | 2 | 2 | 2 | - |  |
| 5014 | 1561 | Ph1 | PF | A | M | 62,7 | 34 | 27,8 | 31,8 | 2 | 3 | 2 | 1 | 1 |  |
| 5164 | 304 | Ph1 | PF | P | L | 60,4 | 29 | 24 | 26,9 | 1 | 1/2 | 2 | 1 | - |  |
| 5166 | 582 | Ph2 | PF | P | - | 40,1 | 29,1 | 23 | 23,7 | 1 | 2 | 2 | 1 | 1 |  |
| 5166 | 398 | Ph2 | PF | P | - | 39,6 | 28,8 | 23,3 | 23,1 | 1 | 2 | 2 | - | - | eroded |

Table S2. Pathologies and measurments on cattle phalanges. PF, Proximal fused; PFg, Proximal fusing. Pex, Proximal exostose; Dex, Distal exostose. Plip, Proximal lipping; Prox, proximal, Dist, distal; Osteo, osteoartrithis. The abbreviations for the measurements are those provided by von den Driesch (1976).

| Context | Sample No | Element | Fusion | A/P | L/M | GL | Bp | SC | Bd | Pex | Plip | Dex | Prox osteo. | Dist osteo. | Comment |
| --- | --- | --- | --- | --- | --- | --- | --- | --- | --- | --- | --- | --- | --- | --- | --- |
| 5014 | 1568 | Ph1 | PF | A | M | 61,9 | 33,3 | 26,1 | 29,6 | 1 | 2 | 1 | 1 | 1 |  |
| 5014 | 1568 | Ph2 | PF | A | - | 39,2 | 32 | 25,5 | 27,5 | 1 | 1 | 2 | 1 | - | eroded |
| 5014 | 1568 | Ph1 | PF | P | L | 63,3 | 30,1 | 24,4 | 30,5 | 2 | 2 | 2 | 1 | 1 |  |
| 5014 | 1568 | Ph1 | PF | P | - | 64,7 | 30,2 | 22,9 | 27,4 | 1 | 2 | 2 | 1 | 1 |  |
| 5014 | 1566 | Ph1 | PF | A | M | 61,6 | 35,5 | 28,3 | 32,5 | 1 | 2 | 2 | 1 | 1 |  |
| 5014 | 1571 | Ph1 | PF | A | M | 61,6 | 32,9 | 26,1 | 30,2 | 1 | 2 | 2 | 1 | 1 |  |
| 5538 | 511 | Ph1 | PF | P | L | 63,8 | 29,5 | 25,2 | 28,3 | - | - | - | - | - | eroded |
| 5418 | 1124 | Ph1 | PF | A | L | 63 | 32,4 | 27,6 | 31,7 | 2 | 2 | 2 | 2 | 1 |  |
| 5418 | 1217 | Ph1 | PF | A | L | 60,4 | 33,8 | 27,3 | 31,4 | 2 | 2 | 2 | 2 | 1 |  |
| 5418 | 970 | Ph1 | PFg | A | L | 62,1 | 27,2 | 21,4 | 26,5 | 2 | 1 | 2 | 1 | 1 |  |
| 5418 | 1204 | Ph1 | PF | A | L | 64,1 | 35,8 | 28,9 | 32,5 | 2 | 2 | 2 | 2 | 1 |  |
| 5418 | 1112 | Ph1 | PF | A | L | 59,8 | 33,2 | 26,4 | 31,1 | 2 | 2 | 2 | 2 | 1 |  |
| 5418 | 1262 | Ph1 | PF | P | L | 65,2 | 36,1 | 29 | 33,8 | 2 | 2 | 2 | 1 | 1 |  |
| 5014 | 1566 | Ph1 | PF | P | M | 62,9 | 33 | 25,8 | 28,8 | 2 | 2 | 2 | 2 | 1 |  |
| 5014 | 1562 | Ph1 | PF | P | L | 69,5 | 33,1 | 25,9 | 28,5 | 2 | 2 | 2 | 2 | 1 |  |
| 5380 | 560 | Ph1 | PF | P | M | 60,7 | 28,3 | 21,9 | 25,1 | 2 | 2 | 2 | 1 | 1 |  |
| 5014 | 1567 | Ph1 | PF | A | - | 65,1 | 37,3 | 29,1 | 33,4 | 2 | 2 | 3 | 2 | 1 |  |
| 5169 | 146 | Ph1 | PFg | A | - | 62,2 | 31,1 | 23,7 | 28,3 | 2 | 2 | 2 | - | - | eroded |
| 5169 | 107 | Ph1 | PF | A | - | 60,9 | 30,8 | 24 | - | - | - | - | - | - | eroded |

Table S2. Pathologies and measurments on cattle phalanges. PF, Proximal fused; PFg, Proximal fusing. Pex, Proximal exostose; Dex, Distal exostose. Plip, Proximal lipping; Prox, proximal, Dist, distal; Osteo, osteoartrithis. The abbreviations for the measurements are those provided by von den Driesch (1976).

| Context | Sample No | Element | Fusion | A/P | L/M | GL | Bp | SC | Bd | Pex | Plip | Dex | Prox osteo. | Dist osteo. | Comment |
| --- | --- | --- | --- | --- | --- | --- | --- | --- | --- | --- | --- | --- | --- | --- | --- |
| 5169 | 147 | Ph1 | PF | A | - | 61,9 | 29,6 | 24,7 | 28,6 | 2 | 2 | 2 | 1 | 1 |  |
| 5503 | 937 | Ph1 | PF | P | - | 62,1 | 28,8 | 23 | 26,9 | 2 | 2 | 2 | 1 | 1 |  |
| 5503 | 467 | Ph1 | PF | P | - | 62,7 | 29,3 | 26,1 | 29 | - | - | - | - | - | eroded |
| 5418 | 1251 | Ph1 | PF | - | - | 68,4 | 32,2 | 26,4 | 30,8 | 2 | 2 | 2 | (1) | 1 |  |
| 5418 | 1242 | Ph1 | PF | P | - | 59,3 | 29,9 | 23,2 | 27,8 | 1 | 2 | 2 | 2 | 1 |  |
| 5014 | 1398 | Ph1 | PF | A | - | 61,3 | 28,9 | 21,7 | 26,1 | 2 | 2 | 2 | 1 | 1 |  |
| 5380 | 1418 | Ph1 | PF | P | L | 63,7 | 32 | 26,4 | 31,1 | 2 | 2 | 2 | 1 | 1 |  |
| 5380 | 1418 | Ph1 | PF | P | L | 70,3 | 30,8 | 25,7 | 29,8 | 2 | 1 | 2 | 1 | - |  |
| 5380 | 1418 | Ph1 | PF | P | L | 63,2 | 30,1 | 25,1 | 28,1 | 2 | 2 | 2 | 1 | 1 |  |
| 5380 | 1418 | Ph1 | PF | P | L | 62,7 | 32,5 | 26,4 | 30,5 | 2 | 2 | 2 | 1 | 1 |  |
| 5380 | 1418 | Ph1 | PF | P | L | 59,9 | 28 | 23,8 | 27,1 | 2 | 2 | 2 | 1 | 1 |  |
| 5538 | 1429 | Ph1 | PF | A | M | 60 | 30,1 | 26,2 | 28,3 | 2 | 1 | 2 | 1 | 1 |  |
| 5538 | 1429 | Ph1 | PF | A | L | 64 | 28,9 | 25 | 27,4 | 2 | 2 | 2 | 1 | 1 |  |
| 5538 | 1429 | Ph1 | PF | A | L | 60,2 | 28,8 | 23,1 | 26,3 | 2 | 2 | 2 | 1 | 1 |  |
| 5418 | 1303 | Ph1 | PF | P | M | 58,1 | 27,2 | 22,7 | 26,9 | 2 | 2 | 2 | 1 | 1 |  |
| 5418 | 1303 | Ph1 | PF | P | M | 60,1 | 32,1 | 25,8 | 29,5 | 2 | 2 | 2 | 1 | 1 |  |
| 5418 | 1303 | Ph1 | PF | P | L | 62,2 | 32,3 | 27,2 | 31 | 2 | 2 | 2 | 1 | 1 |  |
| 5380 | 1417 | Ph1 | PF | A | L | 61,2 | 28,2 | 23,7 | 29,3 | 2 | 2 | 2 | 1 | 1 |  |
| 5380 | 1417 | Ph1 | PF | A | M | 61,1 | 27,9 | 23,2 | 27,1 | 2 | 2 | 2 | 1 | 1 |  |
| 5380 | 1495 | Ph1 | PF | P | L | 62,2 | 28,4 | 23 | 27,5 | 2 | 2 | 2 | 1 | 1 |  |
| 5380 | 1495 | Ph1 | PF | P | - | 62,1 | 31 | 25,3 | 30,8 | 2 | 2 | 2 | 1 | 1 |  |
| 5380 | 1495 | Ph1 | PF | P | M | 63,9 | 28,8 | 24 | 27,2 | 2 | 2 | 2 | 1 | 1 |  |

Table S2. Pathologies and measurments on cattle phalanges. PF, Proximal fused; PFg, Proximal fusing. Pex, Proximal exostose; Dex, Distal exostose. Plip, Proximal lipping; Prox, proximal, Dist, distal; Osteo, osteoartrithis. The abbreviations for the measurements are those provided by von den Driesch (1976).

| Context | Sample No | Element | Fusion | A/P | L/M | GL | Bp | SC | Bd | Pex | Plip | Dex | Prox osteo. | Dist osteo. | Comment |
| --- | --- | --- | --- | --- | --- | --- | --- | --- | --- | --- | --- | --- | --- | --- | --- |
| 5380 | 1418 | Ph1 | PF | P | M | 57,2 | 27 | 22,6 | 26,1 | 2 | 2 | 2 | 1 | 1 |  |
| 5538 | 1382 | Ph1 | PF | A | M | 60,4 | 31,1 | 26,7 | 28,8 | 2 | 1 | 2 | 1 | 1 |  |
| 5538 | 1382 | Ph1 | PF | A | L | 62,1 | 30,7 | 26,1 | 28 | 2 | 2 | 2 | 1 | 1 |  |
| 5014 | 863 | Ph1 | PF | P | M | 61,9 | 29 | 22,1 | 27,3 | 2 | 2 | 2 | 1 | 1 |  |
| 5538 | 1461 | Ph1 | PF | P | - | 61,2 | 30 | 25,7 | 27,6 | 2 | 2 | 2 | 1 | 1 |  |
| 5014 | 380 | Ph1 | PF | A | - | 58,1 | 25,2 | 21,7 | 26,6 | 2 | 2 | 2 | 1 | 1 |  |
| 5325 | 786 | Ph1 | PF | P | - | 66,4 | 32,2 | 27,3 | 29,3 | 2 | 2 | 2 | 1 | 1 |  |
| 5380 | 561 | Ph1 | PF | P | - | 65,3 | (26.5) | 23,3 | 27,8 | 2 | 2 | 2 | 1 | 1 |  |
| 5325 | 692 | Ph1 | PF | P | - | 63,9 | 30,5 | 26,4 | 29,4 | 2 | 1 | 2 | 1 | 1 |  |
| 5014 | 1388 | Ph1 | PF | A | - | 61,2 | 29,5 | 27,2 | 29,4 | 2 | 1 | 2 | 1 | 1 |  |
| 5325 | 705 | Ph1 | PF | P | L | 63 | 28,8 | 23,7 | 27,8 | 2 | 2 | 2 | 1 | 1 |  |
| 5418 | 1439 | Ph1 | PF | P | L | 67,9 | 32,9 | 25,4 | 30,1 | 2 | 3 | 2 | 1 | 1 |  |
| 5538 | 1394 | Ph1 | PF | A | L | 61 | 30,8 | 25,9 | - | 2 | 2 | 2 | 1 | 1 |  |
| 5380 | 1462 | Ph1 | PF | A | L | 62,9 | 29,4 | 25,5 | 28,9 | 2 | 2 | 2 | 1 | 1 |  |
| 5418 | 1062 | Ph1 | PF | P | - | 68,1 | 31,3 | 27,5 | 30,4 | 2 | 2 | 2 | 1 | 1 |  |
| 5538 | 481 | Ph1 | PF | P | L | 63,9 | 28 | 25,6 | 28 | 2 | 2 | 3 | 1 | 1 |  |
| 5014 | 654 | Ph1 | PF | P | M | 60,2 | 30,6 | 24,4 | 27,6 | 2 | 2 | 2 | 1 | 1 |  |
| 5538 | 1391 | Ph1 | PF | P | - | 63,2 | 29 | 25 | 27,2 | 2 | 2 | 2 | 1 | 1 |  |
| 5380 | 1406 | Ph1 | PF | P | L | 59 | 27,1 | 22,4 | 27,1 | 2 | 2 | 2 | 1 | 1 |  |
| 5014 | 884 | Ph1 | PF | A | - | 56,5 | 28,9 | 25,5 | 28,8 | 2 | 2 | 2 | 1 | 1 |  |
| 5014 | 396 | Ph1 | PF | P | - | 60,9 | - | 24,9 | 27,3 | 2 | 2 | 2 | 1 | 1 |  |
| 5014 | 1393 | Ph1 | PF | A | - | 61,8 | 27,4 | 23,5 | 27,4 | 2 | 2 | 2 | 1 | 1 |  |

Table S2. Pathologies and measurments on cattle phalanges. PF, Proximal fused; PFg, Proximal fusing. Pex, Proximal exostose; Dex, Distal exostose. Plip, Proximal lipping; Prox, proximal, Dist, distal; Osteo, osteoartrithis. The abbreviations for the measurements are those provided by von den Driesch (1976).

| Context | Sample | Bone | Bd | e | D1 | e/D1 |
| --- | --- | --- | --- | --- | --- | --- |
| 5538 | 522 | Mc | 61.8 | 19.4 | 26.2 | 0,75 |
| 5014 | 1574 | Mc | 64.0 | 18.8 | 26.0 | 0,72 |
| 5418B | 1217 | Mc | 63.1 | 18.5 | 25.6 | 0,72 |
| 5418B | 1213 | Mc | 69.0 | 21.0 | 28.1 | 0,75 |
| 5014 | 1566 | Mc | 68.7 | 20.6 | 28.9 | 0,71 |
| 5418B | 1112 | Mc | 63.0 | 19.4 | 25.9 | 0,75 |
| 5418 | 1119 | Mc | 64.2 | 17.6 | 26.3 | 0,67 |
| 5164 | 239 | Mt | 59.9 | 18.1 | 24.8 | 0,73 |
| 5325 | 705 | Mt | 55.2 | 16.4 | 23.6 | 0,69 |
| 5418B | 1124 | Mt | 61.3 | 20.4 | 26.2 | 0,78 |
| 5418B | 1214 | Mt | 62.1 | 19.8 | 26.5 | 0,75 |
| 5418B | 505 | Mt | 60.3 | 17.9 | 26.1 | 0,69 |
| 5418 | 1119 | Mt | 64.6 | 23.2 | 27.0 | 0,86 |
| 5503 | 467 | Mt | 57.9 | 16.8 | 23.8 | 0,71 |
| 5418B | 504 | Mt | 56.9 | 17.5 | 23.7 | 0,74 |

Table S3. Measures on metatarsals and traction index calculated following Lin et al (2016): e/D1.

| Context | Sample No | Element | SIDE | P4 | M1 | M2 | M3 | Jones & Sadler (scoring) | Jones & Sadler (absolute age) |
| --- | --- | --- | --- | --- | --- | --- | --- | --- | --- |
| 5418 | 1000 | Mandible | L | d | k | j | g | Gj | 40m-6.5 y |
| 5418 | 1001 | Mandible | R | c | k | g | c | Eb | 34-43 m |
| 5418 | 1001 | Mandible | L | b | k | g | c | Eb | 34-43 m |
| 5164 | 1043 | Mandible | L | b | k | g | b | Eb | 34-43 m |
| 5418 | 1059 | Mandible | R | 0 | k | 0 | c | Eb | 34-43 m |
| 5418 | 1092 | Mandible | L | c | k | g | e | Ee | 34-43 m |
| 5418 | 1093 | Mandible | R | E | k | j | g | Gj | 40m-6.5 y |
| 5418 | 1095 | Mandible | L | c | k | j | g | Gj | 40m-6.5 y |
| 5418 | 1095 | Mandible | R | c | m | k | g | Gk+ | 40m-6.5 y |
| 5014 | 1096 | Mandible | L | A | k | 0 | c | Eb | 34-43 m |
| 5014 | 1096 | Mandible | R | a | k | g | c | Eb | 34-43 m |
| 5418 | 1097 | Mandible | R | c | k | 0 | b | Eb | 34-43 m |
| 5418 | 1097 | Mandible | L | b | k | g | b | Eb | 34-43 m |
| 5418 | 1123 | Mandible | L | 0 | j | k | g | Gk+ | 40m-6.5 y |
| 5014 | 1138 | Mandible | R | 0 | g | b | 0 | Dt | 23-28 m |
| 5014 | 1139 | Mandible | L | 0 | g | b | a | Dt | 23-28 m |
| 5014 | 1202 | Mandible | L | b | j | g | c | Eb | 34-43 m |
| 5418 | 1221 | Mandible | L | e | j | g | b | Eb | 34-43 m |
| 5014 | 1227 | Mandible | L | c | k | 0 | f | F | 34-43 m |
| 5014 | 1227 | Mandible | R | c | 0 | 0 | f | F | 34-43 m |
| 5014 | 1232 | Mandible | R | 0 | h | g | 0 | - | - |
| 5014 | 1232 | Mandible | R | 0 | 0 | 0 | d | Eb | 34-43 m |

Table S4. Eruption and attrition stages of cattle teeth following Grant (1982) and Jones and Sadler (2012).

| Context | Sample No | Element | SIDE | P4 | M1 | M2 | M3 | Jones & Sadler (scoring) | Jones & Sadler (absolute age) |
| --- | --- | --- | --- | --- | --- | --- | --- | --- | --- |
| 5418 | 1296 | Mandible | R | c | k | j | f | F | 34-43 m |
| 5538 | 1382 | Mandible | R | b | k | g | c | Eb | 34-43 m |
| 5538 | 1385 | Mandible | R | b | j | g | c | Eb | 34-43 m |
| 5538 | 1387 | Mandible | L | A | j | g | b | Eb | 34-43 m |
| 5014 | 1388 | Mandible | R | c | k | h | b | Eb | 34-43 m |
| 5325 | 1392 | Mandible | L | A | k | g | b | Eb | 34-43 m |
| 5014 | 1393 | Mandible | L | b | j | g | b | Eb | 34-43 m |
| 5014 | 1402 | Mandible | L | A | g | f | a | Dt | 23-28 m |
| 5325 | 1408 | Mandible | R | A | k | g | b | Eb | 34-43 m |
| 5161 | 141 | Mandible | L | A | k | g | b | Eb | 34-43 m |
| 5765 | 1413 | Mandible | L | 0 | k | 0 | b | Eb | 34-43 m |
| 5765 | 1413 | Mandible | L | 0 | k | c | b | Eb | 34-43 m |
| 5380 | 1418 | Mandible | R | 0 | k | g | a | Dt | 23-28 m |
| 5380 | 1419 | Mandible | L | 0 | 0 | g | g | Ggh | 3.5-5.5 Y |
| 5380 | 1419 | Mandible | R | 0 | k | g | a | Dt | 23-28 m |
| 5161 | 142 | Mandible | R | 0 | k | g | c | Eb | 34-43 m |
| 5380 | 1462 | Mandible | L | 0 | k | g | a | Dt | 23-28 m |
| 5380 | 1478 | Mandible | L | 0 | k | g | b | Eb | 34-43 m |
| 5380 | 1478 | Mandible | R | 0 | k | g | c | Eb | 34-43 m |
| 5161 | 143 | Mandible | U | A | k | h | b | Eb | 34-43 m |

Table S4. Eruption and attrition stages of cattle teeth following Grant (1982) and Jones and Sadler (2012).

| Context | Sample No | Element | SIDE | P4 | M1 | M2 | M3 | Jones & Sadler (scoring) | Jones & Sadler (absolute age) |
| --- | --- | --- | --- | --- | --- | --- | --- | --- | --- |
| 5380 | 1488 | Mandible | L | 0 | g | g | a | Dt | 23-28 m |
| 5014 | 1562 | Mandible | R | g | l | 0 | k | Gk+ | 7-20 y |
| 5014 | 1562 | Mandible | R | g | m | k | k | Gk+ | 7-20 y |
| 5014 | 1565 | Mandible | U | A | j | f | a | Dt | 23-28 m |
| 5014 | 1569 | Mandible | L | c | k | 0 | c | Eb | 34-43 m |
| 5014 | 1570 | Mandible | R | b | k | 0 | c | Eb | 34-43 m |
| 5014 | 1575 | Mandible | L | f | l | g | g | Ggh | 3.5-5.5 Y |
| 5014 | 1576 | Mandible | L | c | k | g | c | Eb | 34-43 m |
| 5418 | 1662 | Mandible | L | 0 | 0 | g | c | Eb | 34-43 m |
| 5164 | 190 | Mandible | R | A | k | f | b | Eb | 34-43 m |
| 5164 | 190 | Mandible | L | a | k | f | b | Eb | 34-43 m |
| 5164 | 252 | Mandible | R | c | k | 0 | c | Eb | 34-43 m |
| 5164 | 252 | Mandible | R | a | k | 0 | b | Eb | 34-43 m |
| 5166 | 305 | Mandible | L | c | k | h | e | Ee | 34-43 m |
| 5166 | 314 | Mandible | R | d | k | g | e | Ee | 34-43 m |
| 5014 | 345 | Mandible | R | 0 | 0 | 0 | b | Eb | 34-43 m |
| 5014 | 345 | Mandible | L | 0 | 0 | 0 | b | Eb | 34-43 m |
| 5014 | 382 | Mandible | R | 0 | 0 | f | b | Eb | 34-43 m |
| 5014 | 382 | Mandible | L | 0 | k | f | b | Eb | 34-43 m |
| 5014 | 383 | Mandible | L | a | k | f | b | Eb | 34-43 m |
| 5014 | 39 | Mandible | R | a | k | g | b | Eb | 34-43 m |
| 5503 | 461 | Mandible | L | c | j | h | g | Ggh | 3.5-5.5 Y |

Table S4. Eruption and attrition stages of cattle teeth following Grant (1982) and Jones and Sadler (2012).

| Context | Sample No | Element | SIDE | P4 | M1 | M2 | M3 | Jones & Sadler (scoring) | Jones & Sadler (absolute age) |
| --- | --- | --- | --- | --- | --- | --- | --- | --- | --- |
| 5503 | 471 | Mandible | R | b | k | f | b | Eb | 34-43 m |
| 5538 | 486 | Mandible | R | 0 | g | A | 0 | Ct | 13-18 m |
| 5538 | 486 | Mandible | L | 0 | g | A | 0 | Ct | 13-18 m |
| 5418 | 494 | Mandible | R | f | k | k | g | Hx | 40m-6.5 y |
| 5418 | 494 | Mandible | L | f | k | k | g | Hx | 40m-6.5 y |
| 5418 | 525 | Mandible | R | 0 | k | h | c | Eb | 34-43 m |
| 5418 | 526 | Mandible | L | 0 | 0 | 0 | c | Eb | 34-43 m |
| 5538 | 529 | Mandible | R | b | l | g | e | Ee | 34-43 m |
| 5538 | 529 | Mandible | L | A | l | g | e | Ee | 34-43 m |
| 5538 | 534 | Mandible | L | c | k | g | f | F | 34-43 m |
| 5380 | 567 | Mandible | L | b | k | 0 | c | Eb | 34-43 m |
| 5166 | 573 | Mandible | R | A | k | 0 | c | Eb | 34-43 m |
| 5166 | 574 | Mandible | L | a | k | j | c | Eb | 34-43 m |
| 5325 | 632 | Mandible | L | 0 | m | l | k | Hx | 7-20 y |
| 5325 | 632 | Mandible | R | 0 | m | l | k | Hx | 7-20 y |
| 5325 | 635 | Mandible | L | A | j | g | a | Dt | 23-28 m |
| 5014 | 654 | Mandible | L | 0 | k | 0 | c | Eb | 34-43 m |
| 5325 | 710 | Mandible | R | c | k | g | f | F | 34-43 m |
| 5325 | 712 | Mandible | L | c | k | g | f | F | 34-43 m |
| 5325 | 715 | Mandible | L | A | k | g | b | Eb | 34-43 m |
| 5325 | 715 | Mandible | R | A | k | g | b | Eb | 34-43 m |

Table S4. Eruption and attrition stages of cattle teeth following Grant (1982) and Jones and Sadler (2012).

| Context | Sample No | Element | SIDE | P4 | M1 | M2 | M3 | Jones & Sadler (scoring) | Jones & Sadler (absolute age) |
| --- | --- | --- | --- | --- | --- | --- | --- | --- | --- |
| 5325 | 717 | Mandible | R | A | k | g | e | Ee | 34-43 m |
| 5325 | 718 | Mandible | L | A | k | g | e | Ee | 34-43 m |
| 5325 | 720 | Mandible | R | c | k | h | c | Eb | 34-43 m |
| 5325 | 722 | Mandible | R | 0 | j | g | b | Eb | 34-43 m |
| 5325 | 722 | Mandible | L | A | j | g | b | Eb | 34-43 m |
| 5325 | 726 | Mandible | L | c | k | h | d | Eb | 34-43 m |
| 5325 | 787 | Mandible | R | 0 | 0 | 0 | b | Eb | 34-43 m |
| 5325 | 787 | Mandible | R | 0 | 0 | 0 | b | Eb | 34-43 m |
| 5418 | 842 | Mandible | R | 0 | k | k | k | Gk+ | 7-20 y |
| 5418 | 842 | Mandible | L | g | k | k | k | Gk+ | 7-20 y |
| 5014 | 853 | Mandible | R | 0 | 0 | 0 | b | Eb | 34-43 m |
| 5014 | 862 | Mandible | L | A | k | g | b | Eb | 34-43 m |
| 5014 | 862 | Mandible | R | A | k | g | b | Eb | 34-43 m |
| 5418 | 885 | Mandible | R | b | j | g | c | Eb | 34-43 m |
| 5418 | 971 | Mandible | R | f | k | j | g | Gj | 40m-6.5 y |

Table S4. Eruption and attrition stages of cattle teeth following Grant (1982) and Jones and Sadler (2012).
